# Prenatal alcohol exposure promotes nerve injury-induced pathological pain following morphine treatment via NLRP3-mediated peripheral and central proinflammatory immune actions

**DOI:** 10.1016/j.bbi.2025.06.041

**Published:** 2025-07-06

**Authors:** Andrea A. Pasmay, Ariana N. Pritha, Justin R. Carter, Alissa Jones, Annette K. Fernandez-Oropeza, Melody S. Sun, Diane C. Jimenez, Minerva Murphy, C.Fernando Valenzuela, Shahani Noor

**Affiliations:** Department of Neurosciences, University of New Mexico HSC, Reginald Heber Fitz Hall-145, MSC08 4740, Albuquerque, NM 87131, United States

**Keywords:** Prenatal alcohol exposure, Glia, Neuroimmune, Allodynia, Peripheral immune, Morphine, NLRP3 inflammasome, Cytokines, Interleukin-1β, MCC950

## Abstract

Adverse in-utero conditions may exert a lifelong impact on neuroimmune function. Our prior work showed that prenatal alcohol exposure (PAE) increases pathological pain sensitivity (allodynia) following peripheral sciatic nerve injury. While the immune mechanism(s) of PAE-induced immune dysfunction are poorly understood, prior studies implicated the involvement of Toll-like receptor 4 (TLR4) and the nucleotide-binding domain, leucine-rich repeat-containing family, pyrin domain-containing 3 (NLRP3) inflammasomes. Interestingly, emerging data suggest a surprising overlap of spinal glial proinflammatory activation via the TLR4-NLRP3-interleukin (IL)-1β axis due to opioid treatment in nerve-injured non-PAE rodents. Considering this preclinical evidence, we explored whether PAE poses a risk factor in creating proinflammatory immune bias consequent to opioid (morphine) exposure. We hypothesized that under nerve injury conditions, PAE may interact with morphine, promoting peripheral and CNS proinflammatory factors in a NLRP3-dependent manner. Using a minor nerve injury model in adult mice, we demonstrate that PAE prolongs the chronicity of ongoing allodynia in both sexes, with a more pronounced effect observed in male mice. Our study shows that PAE amplifies proinflammatory responses at the injury site and the spinal cord, driving morphine-prolonged allodynia through NLRP3 inflammasome activation. Furthermore, high mobility group box 1 (HMGB1), a well-established pain-promoting TLR4 agonist, is elevated in allodynic PAE mice. NLRP3 inhibitor, MCC950, effectively reverses morphine-induced allodynia and reduces Caspase-1 activity, IL-1β, and related proinflammatory factors. Although few sex-specific effects were observed, our data convincingly support that PAE and morphine interactions ultimately converge on NLRP3-driven mechanisms in both sexes. Together, this study suggests that PAE modulates later-life neuroimmune function and provides critical insights into immune regulators underlying PAE-induced biological vulnerability to pathological pain processing and adverse effects of opioids.

## 1. Introduction

Prenatal alcohol exposure (PAE) results in a range of adverse physiological and neurobehavioral outcomes, recognized as fetal alcohol spectrum disorders (FASDs) (Popova et al., 2021; Sn et al., 2019). FASDs occur worldwide and are as high as 5 % in the US, estimated to be more frequent than other neurodevelopmental disorders, including autism (Sn et al., 2019). Notably, sensory processing issues (Ab et al., 2020; Fjeldsted and Xue, 2019; Saha and Mayhan, 2022), including touch hypersensitivity, are common in individuals with FASD. Consistent with clinical observations, numerous preclinical studies indicate that PAE induces biological vulnerability to lifelong dysfunctional central nervous system (CNS)-immune interactions (Sn et al., 2019; Noor and Milligan, 2018) heightens proinflammatory neuroimmune activity upon exposure to subsequent immune stimuli (Ls and Jm, 2016), although key immune signaling pathways involved are poorly understood. In recent years, we have utilized an adult-onset peripheral nerve injury model to unmask PAE-induced susceptibility to chronic CNS dysfunction leading to pathological touch sensitivity (allodynia) (Fjeldsted and Xue, 2019; Breuer et al., 2024).

Mechanical allodynia is a common clinical symptom of chronic pain. Chronic allodynia results from heightened excitation of spinal cord pain neurons due to aberrant neuronal-glial immune interactions (Gwak et al., 2017); often consequent to peripheral nerve injury (Staikopoulos et al., 2021). Following nerve injury, endogenous cell stress factors (damage-associated molecular patterns, DAMPs) e.g., HMGB1, are released that, in turn, activate the immune receptor Toll-like receptor 4 (TLR4) on peripheral immune and spinal glial cells (Relja and Land, 2020; Yang et al., 2021; Wan et al., 2016). TLR4 activation leads to translocation of the transcription factor, NF-kB, inducing the transcription of proinflammatory factors, including pro-IL-1β, pro-IL-18, TNF-α and nod-like receptor family pyrin domain containing 3 (NLRP3). NLRP3 further assembles with other proteins to form the NLRP3 inflammasome complex that activates Caspase-1, a necessary step for the production and release of a mature form of IL-1β (He et al., 2016) and IL-18 (Alboni et al., 2010; Begum et al., 2024; Ju et al., 2024). Proinflammatory immune activation in the periphery results in the sensitization of nociceptors, resulting in increased excitability and peripheral sensitization (Cui et al., 2023; Ohashi et al., 2023). This enhanced nociceptive input is relayed to the spinal cord, where it activates glial cells and induces local inflammatory cascades, promoting central sensitization (Bhatt et al., 2024; Milligan et al., 2003). Recent studies also highlight the involvement of the TLR4-NLRP3 axis in supraspinal regions, particularly in the midbrain periaqueductal gray (PAG) and the anterior cingulate cortex (ACC), which play critical roles in pain processing (Ko et al., 2019; Guo et al., 2021; Napadow et al., 2019; Yao et al., 2019); reinforcing proinflammatory neuroimmune signaling along the pain pathway (Guo et al., 2021; Bruno et al., 2018). Together, persistent TLR4-NLRP3-driven signaling cascade across the periphery, spinal cord, and brain contributes to the development and maintenance of mechanical allodynia (Bruno et al., 2018; Chen and Smith, 2023).

Beyond their crucial neuroimmune modulatory roles during chronic allodynia, glia also play a significant role in influencing opioid tolerance and hyperalgesia associated with opioid pain therapeutics (Wang et al., 2024; Holdridge et al., 2007; Hutchinson et al., 2007; Watkins et al., 2005). While opioids, such as morphine, primarily act on the excitability of the pain neurons via mu-opioid receptors, inducing pain relief, opioid-induced proinflammatory actions via the TLR4-NLRP3-IL-1β pathway have been reported (Zhang et al., 2020). In fact, proinflammatory effects from repeated morphine on primed immune cells (Gessi et al., 2016) can act as an exogenous TLR4 activator underlying opioid-induced tolerance, and hyperalgesia observed clinically and in numerous preclinical pain models (Zhang et al., 2020; Ellis et al., 2016; Grace et al., 2016). While most preclinical studies examine morphine’s effects within hours of treatment, morphine paradoxically prolongs nerve injury-induced allodynia in male rats *after discontinuation* of treatment (Grace et al., 2016; Green-Fulgham et al., 2019). While these studies highlighted the involvement of TLR4 and NLRP3 activation on spinal glia (Grace et al., 2016), potential sex differences and the effects of opioids modulating these immune factors in the periphery and brain regions remain unknown.

Our prior work suggests a potential overlap between neuroimmune pathways in morphine-mediated actions on glial cells (Berta et al., 2012) and PAE-induced changes in neuroimmune function (Noor and Milligan, 2018; Noor et al., 2020; Sanchez et al., 2017) during adulthood. Notably, PAE rodents exhibit chronic allodynia following minor nerve injury, whereas non-PAE rodents subjected to the same injury do not (Noor et al., 2020; Sanchez et al., 2017; Sanchez et al., 2019; Noor et al., 2019). PAE-related susceptibility to chronic allodynia was associated with enhanced IL-1β and TNFα at the peripheral nerve injury site (Noor et al., 2017) and spinal cord (Sanchez et al., 2019). Our recent report explored the potential overlap of PAE-induced neuropathic pain susceptibility and morphine-mediated actions in female PAE mice (Noor et al., 2023). A striking observation was made suggesting that following minor nerve injury, the chronicity of allodynia is further exacerbated from a 5-day regimen of morphine treatment in PAE mice, whereas non-PAE control mice did not show allodynia. Moreover, utilizing a well-established small molecule inhibitor of NLRP3, MCC950 (Tapia-Abellán et al., 2019; Chen et al., 2021; Li et al., 2022; Jiao et al., 2020; Perera et al., 2018; Xue et al., 2022; Shen et al., 2022) we found that prolonged allodynia following morphine treatment was NLRP3-dependent. However, a comprehensive understanding of the molecular changes and potential sex differences related to TLR4 and NLRP3 signaling that drive the prolongation of allodynia under PAE conditions is yet to be explored.

Here, utilizing a previously characterized model of a minor sciatic nerve injury (Noor et al., 2017), we hypothesized that PAE interacts with morphine to promote peripheral and CNS proinflammatory factors, driven by NLRP3 inflammasome activation. To test this hypothesis, we: 1) Investigated whether PAE-induced susceptibility to morphine-prolonged allodynia occurs in both male and female mice; a side-by-side comparison characterizing the onset, duration and spontaneous reversal of allodynia was conducted. 2) Performed behavioral assessments of allodynia, with or without MCC950-treated mice, to confirm the necessary role of NLRP3 in morphine-induced prolonged allodynia in both sexes. 3) Examined molecular changes associated with the TLR4-NLRP3 axis in the pain-relevant PNS (sciatic nerve) and CNS (lumbar spinal cord and brain) regions.

## 2. Materials and methods

### 2.1. Animals

All procedures were approved by the Institutional Animal Care and Use Committee (IACUC) of the University of New Mexico (UNM) Health Sciences Center. Animal experiments adhered to ARRIVE guidelines and the National Institutes of Health guide for laboratory animal care and use (NIH Publications No. 8023, revised 1978). All mice were routinely monitored by the animal care staff under the direction of the institutional veterinarian and staff, with cages and bedding changed every 7 days. All behavioral assessments, injections, and tissue collections were performed within the first 2 h of the inactive cycle (light cycle) to avoid the influence of endogenous circadian regulation in the production of proinflammatory cytokines.

### 2.2. Prenatal alcohol exposure (PAE) paradigm

A well-established paradigm for generating experimental moderate prenatal alcohol-exposed offspring was utilized (Lm et al., 2013; Lm et al., 2012). PAE or age-matched control mice were provided at weaning by the New Mexico Alcohol Research Center (NMARC). Briefly, C57BL/6J mice were obtained from The Jackson Laboratory (Bar Harbor, ME) for breeding. Upon arrival, sires and dams were acclimated in a colony on a 12:12-hour reverse light/dark schedule and fed Teklad 2920X rodent chow and tap water, available *ad libitum*. On day 7, dams are isolated; on days 14–17, dams are exposed to 0.066 % (w/v) saccharin (sac offspring, non-PAE control mice) or 5 % w/v ethanol sweetened with 0.066 % (w/v) sac solution (PAE offspring) for 4hr/day (1000 to 1400 hr). On day 18, dams are switched to 10 % w/v ethanol sweetened with 0.066 % (w/v) sac solution (PAE offspring). For breeding, individual females were placed into a cage with a single-housed male for two hours (from 1400 to 1600 hr) for three consecutive days. Pregnancy was positively determined by monitoring weight gain every 3–4 days. Alcohol exposure occurs throughout pregnancy, and this protocol produces blood EtOH concentrations of about 80 mg/dl following the drinking period in dams, with no negative effects on pup survival, weight, or litter size (Lm et al., 2013). One day after birth, access to alcohol was withdrawn using a step-down procedure, as described previously (Lm et al., 2012). saccharin (sac) (control mice) and PAE offspring were weaned at ~ 3 weeks and subsequently maintained in groups of 2–4 mice per cage. Adult (7–8 months old) female & male PAE or age-matched sac (control) mice offspring were used for all experiments. None of the experimental groups contained more than two subjects from a given litter to avoid “litter effects.” Offspring were habituated to a standard light/dark cycle (lights on from 0600 h to 1800 h) for at least 3 weeks and kept in these conditions for the duration of the study.

### 2.3. Minor chronic constriction injury (CCI)

Mice were exposed to minor nerve injury (minor CCI) with a single suture (6–0 chromic gut) tied around the sciatic nerve, which is a modification of the previously well-established standard chronic constriction injury that involves more robust sciatic nerve damage, with three segments of 4–0 or 5–0 sutures (Noor et al., 2023). Following isoflurane anesthesia (induction at 1.5 %–2.5 vol% followed by 2.0 vol% in oxygen), the dorsal left thigh was shaved and cleaned using 80 % EtOH. The left sciatic nerve is gently exposed using blunt dissection scissors and isolated with sterile plastic probes. A single, 6–0 chromic gut suture (Ethicon, Cat #: 796G) is tied around the sciatic nerve. The suture is composed of a double knot located at the middle of the nerve without pinching the nerve to avoid nerve damage. The nerve was kept moist using isotonic sterile saline. Sham surgeries consist of an exposure of the nerve without suture ligation. The nerve is carefully placed back into its position, and the overlying muscle is sutured using a 4–0 silk suture. The skin was closed using three Reflex^™^ wound clips (Kent Scientific Corp.; Cat #: INS750344). The total time for the surgical procedure was ~ 15 min, followed by a ~5-minute recovery from anesthesia. After the surgical procedure, the healing of the wound, hind paw autotomy, activity levels, and grooming appearance were assessed twice a day for three days to ensure a smooth and healthy recovery process. Staples were removed 10–12 days after surgery, at which point the wound had completely closed. No mice were excluded due to surgical complications.

### 2.4. Behavioral assessment of mechanical allodynia

Behavioral assessment of mechanical allodynia was performed using the Von Frey Fiber test, as described in our prior reports (Noor et al., 2020; Sanchez et al., 2019; Noor et al., 2019). Mice were gently handled and habituated to the testers and the testing environment. Mice were tested in the same room where they are housed, limiting environmental variation. Mice were habituated to the testing environment for ~ 60 min over 4 days before baseline assessment. The Von Frey test involves using nine calibrated monofilaments, which are applied for up to 3.0 s on the plantar surface of both the left and right hind paws. The testing of hind paw laterality is randomized, and the intertrial stimulus interval was at ~ 10 s. Each paw undergoes a maximum of seven stimulus presentations. Lifting, jumping, licking, or shaking the paw was considered a positive response. The total number of positive and negative responses was then entered into the PsychoFit software to determine the absolute withdrawal threshold (50 % paw withdrawal threshold). The PsychoFit program fits a Gaussian integral psychometric function to the observed withdrawal rates for each monofilament using a maximum-likelihood fitting method (Milligan et al., 2000). Cohorts of 6–8 mice were tested, with experimenters unaware of the experimental conditions. Each group consisted of at least 1–2 mice per treatment condition. Time points were carefully selected based on prior studies to capture allodynia’s development, persistence, and resolution (Sanchez et al., 2017). Behavioral evaluations were conducted at baseline (BL), post-surgery days 3, 7, 10, 14, and then every 2–4 days following morphine injection and 90 min and 1 day post MCC950 treatment.

### 2.5. Morphine treatment

Single subcutaneous (Sub-Q) injections of morphine were given for five consecutive days starting on D14 through D18 post-CCI. The timeline for morphine treatment was based on our previous characterization demonstrating that PAE mice reliably develop allodynia following minor nerve injury (Noor et al., 2023), with peak and stable mechanical allodynia occurring around D14. Injections were performed at the same time each day to ensure consistency and minimize variability. The analgesic isomer of morphine, (−) – morphine, was purchased from Sigma, MO, USA (Morphine sulfate salt pentahydrate, Cat #: M8777). Either morphine or vehicle (sterile saline) was injected at a dose of 10 mg/kg body weight, which is considered a moderate dose of morphine (Farsi et al., 2013; Gades et al., 2000).

### 2.6. NLRP3 inhibitor, MCC950, treatment

MCC950, a small molecule inhibitor, was used to assess the role of NLRP3 in the development of morphine-prolonged allodynia. MCC950 inhibits NLRP3 oligomerization by preventing the assembly of ASC (apoptosis-associated speck-like protein) to the NLRP3 complex (Tapia-Abellán et al., 2019) and the release of mature IL-1β (Matsuoka et al., 2020). To date, numerous *in vivo* and *in vitro* studies have validated that MCC950 crosses the blood–brain barrier (Martínez-Martel et al., 2025) and specifically blocks the NLRP3 inflammasome activation, but not other inflammasomes that may induce Caspase-1 (Coll et al., 2015). Moreover, MCC950 has been successfully used to reverse chronic allodynia in numerous preclinical models, without affecting normal nociception (Chen et al., 2019; Chen et al., 2022; Martínez-Martel et al., 2025; He et al., 2019). Using aseptic procedures, a single intraperitoneal injection of MCC950 (10 mg/kg) or vehicle injection was given to unanesthetized mice. The MCC950 dose was chosen based on prior in vivo studies demonstrating reduced neuroinflammation (Jiao et al., 2020; Matsuoka et al., 2020; Zeng et al., 2021). The total time required for handling and injection procedure was under 1 min.

### 2.7. Tissue collection for RNA and protein analysis

Immediately after the last behavioral assessments (D24), tissues were collected for molecular analysis. The mice were induced into deep anesthesia using isoflurane (10 min at 5 vol% isoflurane in oxygen at a 2.0 vol%, then underwent rapid transcardial perfusion with ice-cold 0.1 M phosphate-buffered saline (PBS; pH = 7.4; flow rate 10 mL/min). Blunt dissection scissors were used to re-expose the injured sciatic nerve (SCN, left side). Approximately 1 cm of the SCN was collected, keeping the suture (lesion site) in the middle. Spinal cord was gently flushed out from the vertebral column with ice-cold sterile PBS into a petri dish and ipsilateral lumbar spinal cord dorsal horn LSC (L3-L6) was dissected. The brain was carefully removed from the skull. Midbrain was dissected from the entire brain and the anterior cingulate cortex was dissected from the contralateral (right) hemisphere. All tissues were immediately flash-frozen in dry ice in DNase/RNase/Protease-free 1.5 mL Eppendorf tubes (VWR International; Cat #: 20170–038) and stored at − 80 °C for future analysis.

### 2.8. Total RNA & protein isolation

Tissues were processed for RNA and protein analysis, as described previously (Noor et al., 2019; Noor et al., 2017). Tissues were homogenized with sterile RNAase-free PBS using a motorized VWR disposable pellet mixer and cordless motor pestle system. Homogenized tissue was divided by volume to a ratio of 60:40 and used for RNA and protein analysis, respectively. RNA extraction was performed using QIAzol Lysis Reagent and the miRNeasy Micro Kit (Qiagen; Cat #: 74004), per the manufacturer’s instructions. The concentrations and quality of the total RNA were assessed using NanoDrop (Thermo Scientific, MA, USA). Tissue homogenates designated for protein analysis were preserved in a protein buffer containing a protease inhibitor (VWR; Cat #: 7844) and lysis buffer (Thermo Scientific; Cat #: P187787). The tissue samples were homogenized, sonicated, and centrifuged at 14,000g for 10 min to separate pellet debris from the protein lysate. Per the manufacturer’s instructions, the total protein concentration was determined using a Bradford Assay (Bio-Rad Cat #: 5000201).

### 2.9. High-sensitivity IL-1β ELISA protein assay

Protein concentration was determined using the Quick Start Bradford Protein Assay (Bio-Rad, USA, Cat #: 5000201). IL-1β protein was detected using a high-sensitivity commercial enzyme-linked immunosorbent assay kit (HS Quantikine; R&D Systems, USA, Cat #: MHSLB00) according to the manufacturer’s instructions. IL-1β concentrations were calculated against the internal standards and normalized to the total protein concentration in the homogenate (pg/mL). Due to low protein yield achieved from designating each tissue sample for both protein and mRNA analysis, some samples were pooled from multiple biological replicates and few samples were only designated for protein analysis. For the spinal cord IL-1β assay, a total of 100 μg (females) or 65 μg (males) protein was used. For the sciatic nerve IL-1β 90 μg of total protein was used in both males and females.

### 2.10. Caspase-1 activity assay

Caspase-1 activity is an immediate downstream effect of NLRP3 inflammasome activation. Colorimetric detection of Caspase-1 activity assay was performed according to the manufacturer’s instructions (Abcam Cat #: ab273268). Most quantitative molecular assays detect both pro-IL-1β and mature forms of IL-1β protein from tissue lysates. Caspase-1 activity assay utilizes the activity of Caspase-1 that recognizes the sequence YVAD, hence providing a reliable readout of NLRP3 inflammasome activation and production of mature IL-1β (Barreiro-Iglesias and Shifman, 2012). Briefly, protein samples were measured by Bradford Assay, and 60–65 μg total protein was incubated with 5 μl of YVAD-p-nitroanilide (pNA) and incubated at 37 °C for 2 h. The activity of Caspase-1 is measured by spectrophotometric detection of the chromophore p-nitroanilide (pNA) at 400 nm after cleavage from the labeled substrate YVAD-pNA. For spinal cord tissue, all experimental groups were included for females, but due to the limited protein amount left from male tissues, additional mice were added to the study, with only the three critical groups.

### 2.11. mRNA analysis by quantitative Real-Time PCR

Relative mRNA levels were measured as described in our prior reports (Noor et al., 2019). Briefly, total RNA samples were diluted to a standardized RNA concentration, and their concentrations and quality were further confirmed by NanoDrop measurement. Total RNA (1.0–1.2 μg) was used to synthesize reverse transcription (cDNA). For cDNA synthesis, SuperScript^™^ IV VILO^™^ cDNA Synthesis Kit (Thermo Fischer Scientific, Cat #: 11754050) was used per the manufacturer’s instructions. A 1:2.5 dilution factors were applied to cDNA samples for assessment of transcripts of interest in the ipsilateral spinal cord, ipsilateral sciatic nerve, contralateral ACC, and whole midbrain. The 1:200 dilutions of cDNA were used to assess the normalizer transcripts (18 s RNA) for each tissue sample. All samples were assayed in triplicates via quantitative real-time PCR (qRT-PCR) with TaqMan Gene Expression Assays (Noor et al., 2019). Standard deviations were calculated for triplicate measurements, and when exceeding 0.1, the average value of the two closest replicates was utilized. Relative gene expression levels were analyzed based on the endogenous controls (normalizer, 18 s), and data are presented as fold increases relative to the non-allodynic control group (sac + minor CCI + veh + veh), using the formula: 2^−ΔΔ^CT (Livak and Schmittgen, 2001). The following pain-relevant proinflammatory transcripts were evaluated: high mobility group box 1 (HMGB1, *hmgb1*) interleukin-1β (IL-1β, *il1b*), interleukin-18 (IL-18, *il18*) tumor necrosis factor α (TNFα, *Tnf*), NOD-like receptor protein-3 (NLRP3, *nlrp3*), IkappaB alpha (IKBA. *lkBa*), GFAP (*Gfap*), an astrocyte activation marker, and Iba-1 (*Aif-1*), a marker for microglia activation. The μ-opioid receptor is known to play a crucial role in modulating pain signaling pathways (Al-Hasani and Bruchas, 2011). Although it is beyond the scope of this study to examine activation of the u-opioid receptor from morphine, expression of the μ-opioid receptors was measured to examine potential PAE-related effects. Samples from male and female mice were processed simultaneously to minimize variability.

### 2.12. Experimental design

**Fig. 1.**
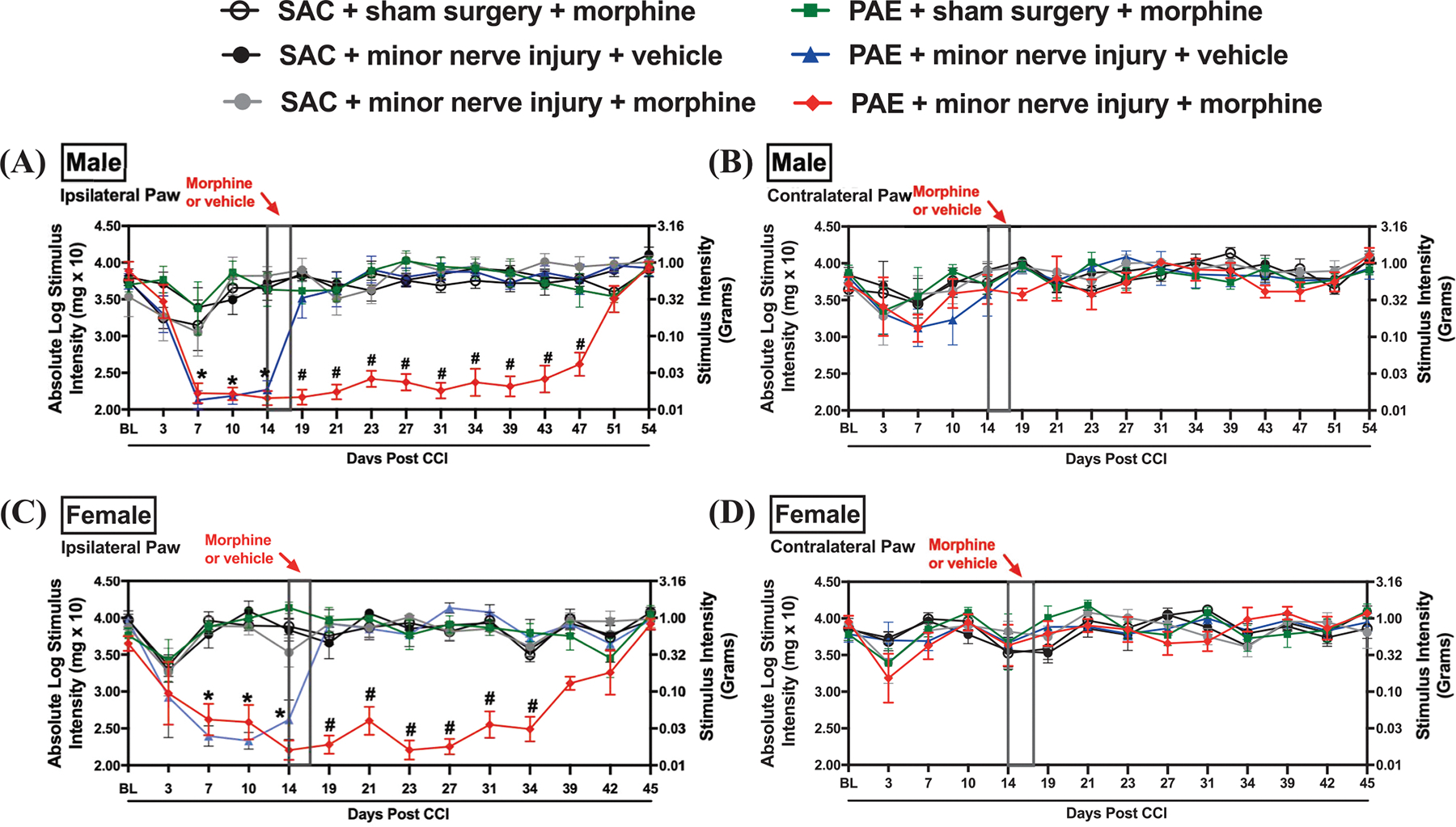
Morphine treatment prolongs the duration of allodynia only in minor nerve-injured PAE mice in both sexes. Sac and PAE mice of both sexes were exposed to sham surgery or minor nerve injury (CCI), and the effects of morphine treatment on hind paw sensitivity were examined. Pre-surgery baseline (BL) values were similar in sac and PAE mice (F_1,54_ = 0.48, p = 0.49). Ipsilateral hind paw sensitivity developed following minor CCI. (A & C) A significant interaction between PAE × time (F_3,150_ = 7.06, p = 0.00002), PAE × surgery (F_1,50_ = 104.71, p < 0.0001), and PAE × surgery × time (F_3,150_ = 4.03, p = 0.009) was observed only on the ipsilateral side. In males and females, nerve-injured PAE mice displayed a significant increase in ipsilateral hind paw sensitivity by D7–D14 (p < 0.05). (B & D) In both sexes, no contralateral allodynia was observed post-minor nerve injury. Post-morphine hind paw responses were evaluated between the PAE morphine-treated and vehicle-treated mice. (A) Post-morphine treatment, there was a significant interaction of PAE × morphine (F_1,29_ = 182.39, p < 0.0001). In males, morphine-injected PAE mice showed persistent ipsilateral hind paw sensitivity compared to vehicle-treated PAE mice up to D47 post-CCI (D23–D47; # p < 0.04). (C) Female PAE mice showed persistent ipsilateral hind paw sensitivity compared to vehicle-treated PAE mice up to D39 post-CCI (D19–D39; # p < 0.0001). (A & C) Morphine-injected PAE mice exhibited allodynia, in contrast to morphine-treated sac mice, which maintained baseline levels throughout the entire time course. (B & D) No significant changes were observed in the contralateral paw. Large effect sizes were observed for PAE exposure (${\text{η}}_{\text{P}}^{2}=0.870$), morphine treatment (${\text{η}}_{\text{P}}^{2}=0.869$), and their interaction (PAE × morphine; ${\text{η}}_{\text{P}}^{2}=0.875$), indicating that PAE mice treated with morphine developed significant allodynia compared to both sac morphine-treated and PAE vehicle-treated mice.

**Fig. 2.**
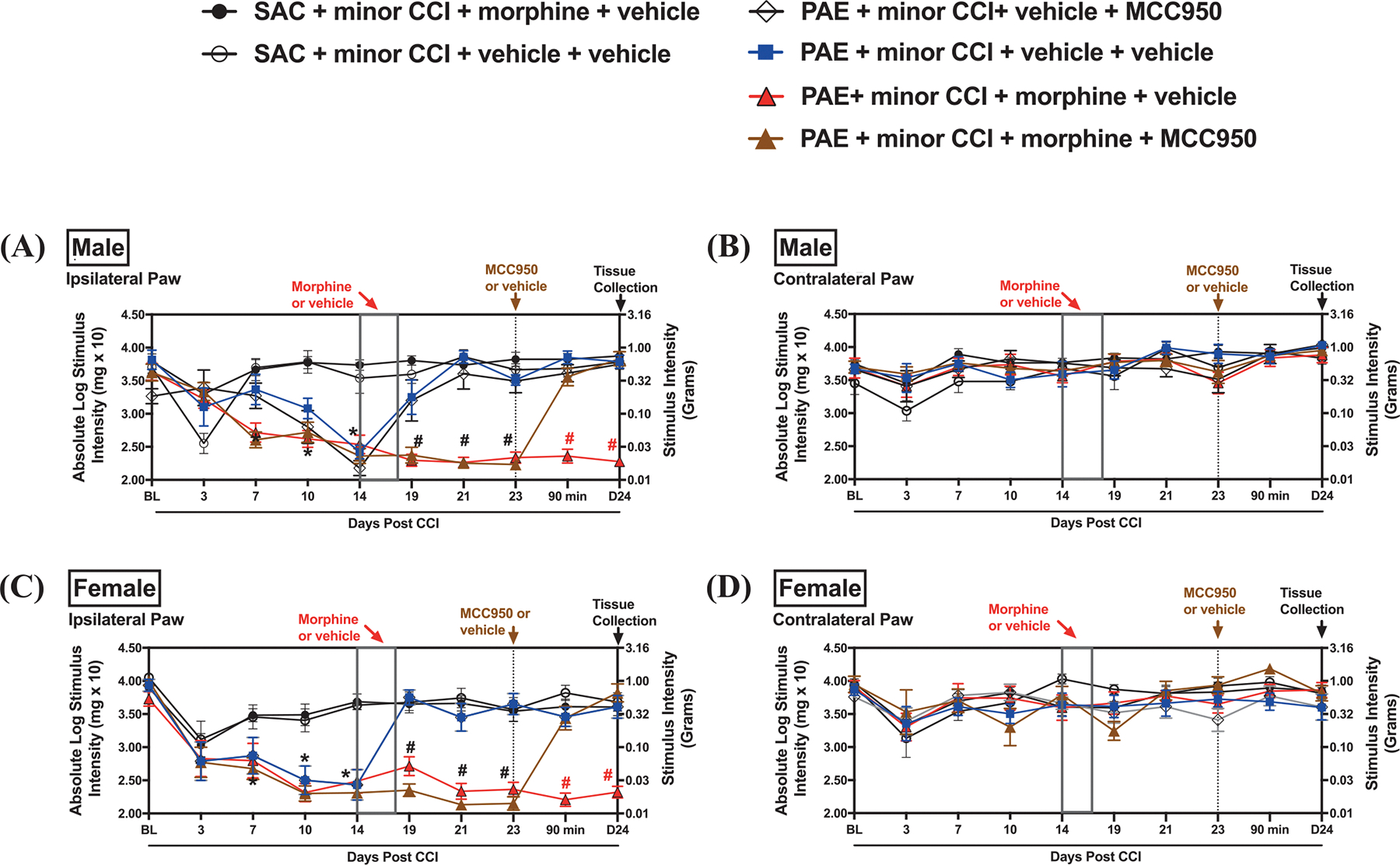
Systemic treatment of NLRP3 inhibitor, MCC950, reverses morphine-mediated prolonged allodynia in PAE mice, regardless of sex. The effects of systemic MCC950 treatment were examined during morphine-prolonged allodynia in nerve-injured PAE mice. A two-way ANOVA revealed no significant differences in pre-surgery (BL) values (F_1,91_ = 2.9, p = 0.09). Following CCI, only PAE mice developed morphine-prolonged allodynia, as indicated by a significant PAE interaction (F_1,364_ = 11.17, p = 0.0009). Male (A) and female (C) PAE mice developed allodynia in comparison to sac mice post-CCI at D7–D14 (p < 0.003). (A & C) Post-morphine treatment, there was a significant interaction of PAE × morphine (F_1,86_ = 132.77, p < 0.0001), as well as a sex × time interaction (F_2,172_ = 4.17, p = 0.0170). Morphine-treated PAE mice exhibited prolonged allodynia on D19–D23 (# p < 0.0001) compared to PAE vehicle-treated mice for both males and females. Additionally, regardless of sex, significant differences in allodynia were observed between morphine-treated PAE mice and morphine-treated sac mice at D19–D23 (p < 0.04). (B & D) Morphine treatment did not result in any changes in the contralateral hind paw. (A & C) There was a significant sex effect (F_1,60_ = 240.3, p < 0.0001) when evaluating the effects of MCC950. In both males and females, MCC950 treatment decreased sensitivity in morphine-treated PAE mice compared to vehicle-treated morphine-treated PAE mice, beginning as early as 90 min post-injection, with reversal continuing 24 h post-injection (# p < 0.0001), at which time tissues were collected. (B & D) Regardless of sex, MCC950 did not alter hind paw responses in the absence of injury on the contralateral side.

**Fig. 3.**
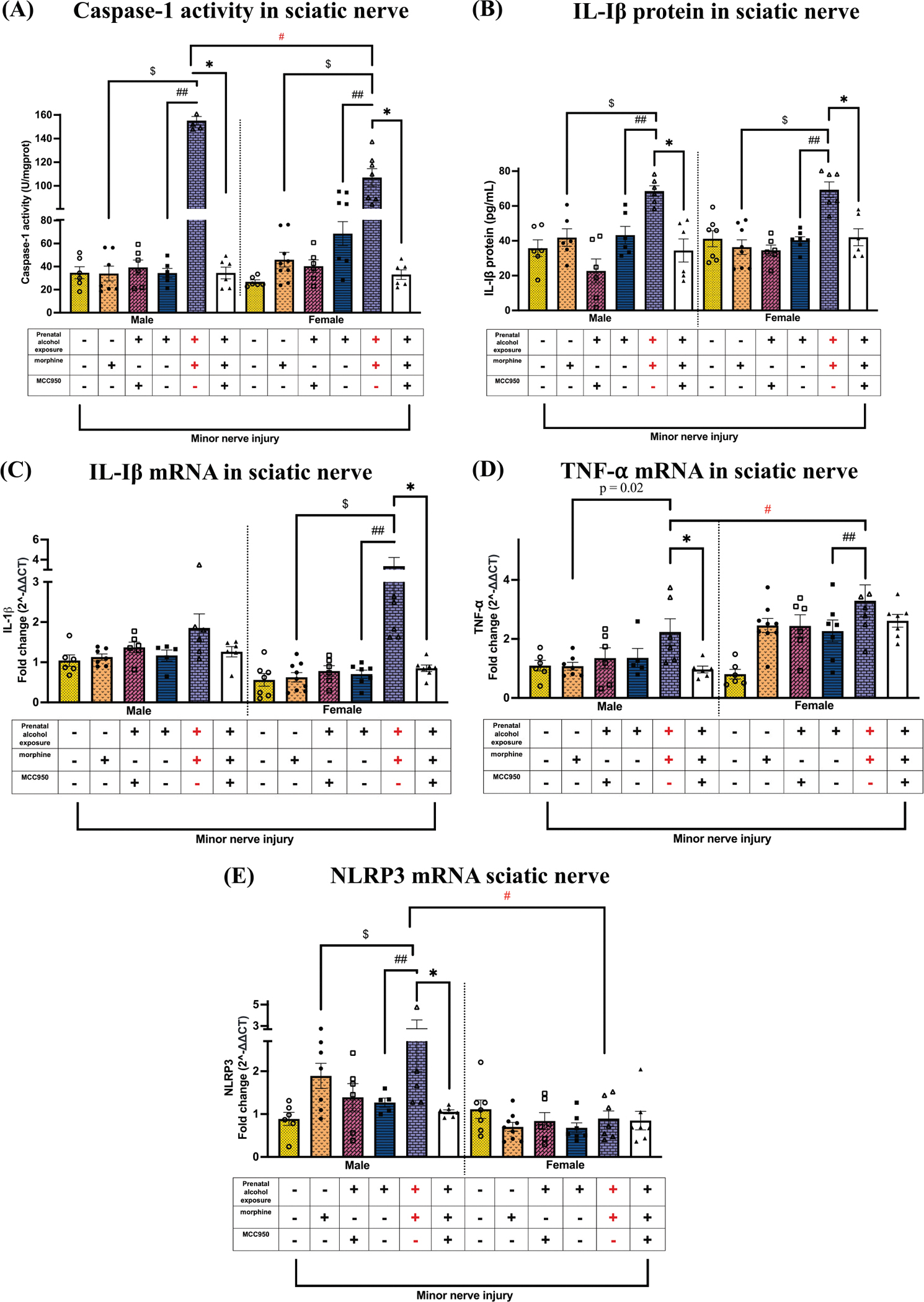
Effects of PAE and morphine immune interactions, and MCC950 treatment on Caspase-1, IL-1β, NLRP3, and TNF-α levels at the injured nerve. Ipsilateral sciatic nerves were collected from behaviorally verified mice, as represented in Fig. 2. Prenatal exposure (+), morphine (+), and MCC950 (−) in purple denote the allodynic group; all other groups are non-allodynic or allodynia-reversed at this post-CCI time point. (A) Despite all mice being exposed to minor nerve injury, morphine treatment significantly increased caspase-1 activity regardless of sex in PAE mice compared to the two key control groups: sac male mice ($ p < 0.0001, PAE vs. sac, morphine-treated mice) and PAE mice without morphine treatment (## p < 0.0001, PAE, morphine vs. vehicle). Post hoc comparisons revealed that male PAE morphine-vehicle mice exhibited significantly higher values compared to their female counterparts (# p < 0.0001). In both males and females, MCC950 treatment reduced caspase-1 activity in morphine-treated PAE mice that displayed allodynia reversal (* p < 0.0001). (B) Despite all mice being exposed to minor nerve injury, morphine treatment significantly increased IL-1β protein regardless of sex in PAE mice compared to the two key control groups: sac male mice ($ p < 0.0009, PAE vs. sac, morphine-treated mice) and PAE mice without morphine treatment (## p < 0.004, PAE, morphine vs. vehicle). In males, MCC950 treatment reduced IL-1β protein in morphine-treated PAE mice that displayed allodynia reversal (* p < 0.04). (C) In females, morphine-prolonged allodynia coincided with a significant increase in IL-1β mRNA in PAE females compared to sac female mice ($ p < 0.0001, PAE vs. sac, morphine-treated mice) and PAE mice without morphine treatment (## p < 0.0001, PAE, morphine vs. vehicle). MCC950 treatment reduced IL-1β mRNA in morphine-treated PAE females that displayed allodynia reversal (* p = 0.01). (D) In males, morphine-prolonged allodynia resulted in a significant increase in TNF-α mRNA levels exclusively in PAE mice treated with morphine compared to sac mice treated with morphine (p = 0.003, unpaired *t*-test). Morphine-prolonged allodynia resulted in a significant increase in TNF-α mRNA levels in PAE female mice treated with morphine compared to PAE females treated with vehicle (## p = 0.04, PAE, morphine vs. vehicle). MCC950 treatment reduced TNF-α mRNA in morphine-treated male PAE mice that displayed allodynia reversal (* p = 0.03). We observed significant sex differences in TNF-α mRNA levels, with females exhibiting higher levels than males in allodynic, morphine-treated PAE mice (# p = 0.04). (E) In male mice, morphine treatment significantly increased NLRP3 mRNA in PAE mice compared to the two key control groups: sac male mice ($ p = 0.05, PAE vs. sac, morphine-treated mice) and PAE mice without morphine treatment (## p = 0.002, PAE, morphine vs. vehicle). NLRP3 mRNA was significantly higher in allodynic male morphine-treated PAE mice compared to their female counterparts (# p < 0.0001). MCC950 significantly reduced NLRP3 mRNA only in morphine-treated male PAE mice that displayed allodynia reversal (* p = 0.007).

**Fig. 4.**
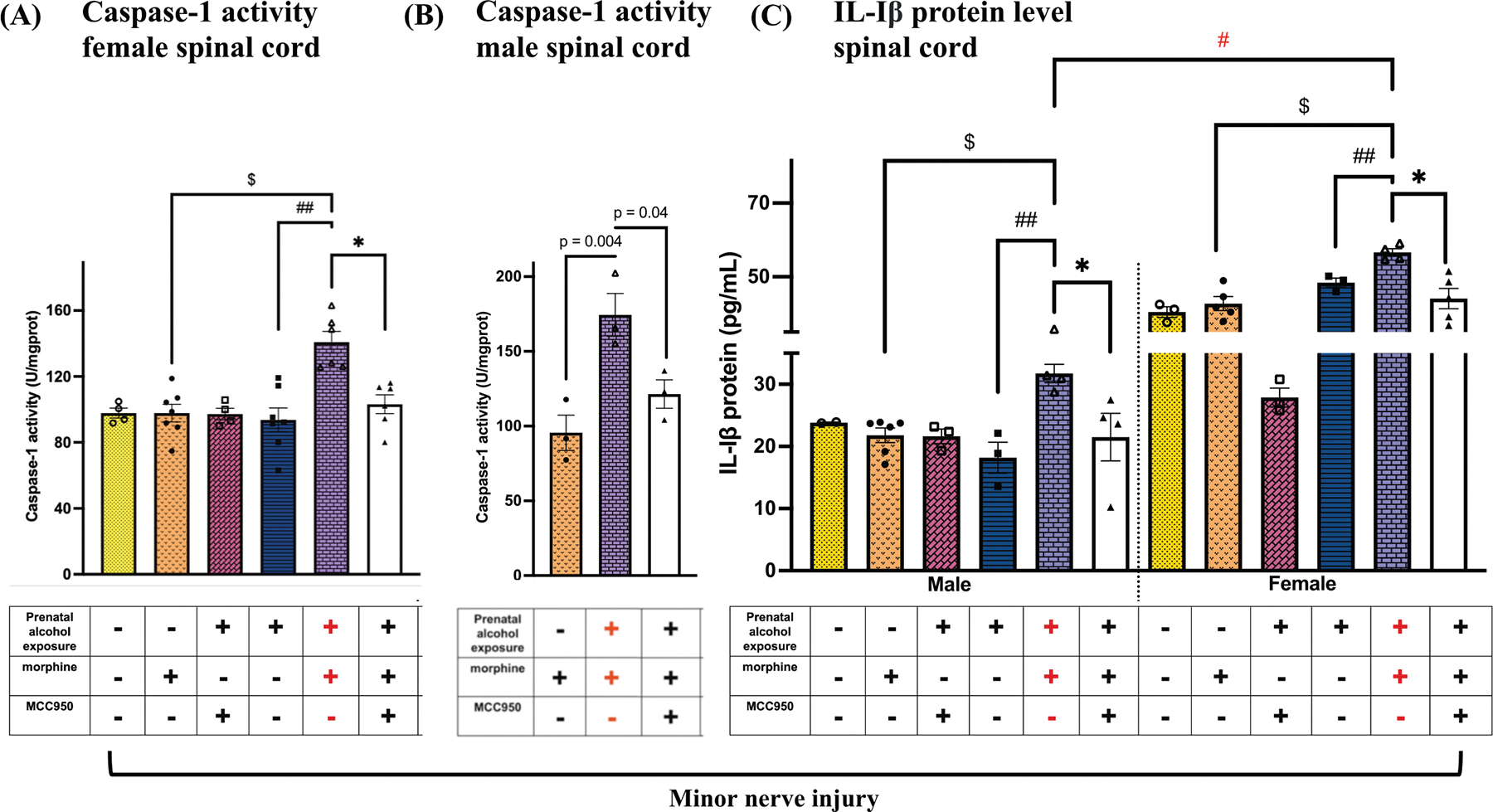
Effects of PAE and morphine immune interactions, and MCC950 treatment on Caspase-1 and IL-1β protein in the spinal cord from minor nerve-injured mice. Spinal cords were collected from behaviorally verified mice, as represented in Fig. 2. Prenatal exposure (+), morphine (+), and MCC950 (−) in purple denote the allodynic group; all other groups are non-allodynic or allodynia-reversed at this post-CCI time point. (A) Despite all female mice being exposed to minor nerve injury, morphine treatment significantly increased spinal Caspase-1 activity in PAE compared to sac mice ($ p < 0.0001, PAE vs. sac, morphine-treated mice) and compared to PAE mice without morphine treatment (## p < 0.0001, PAE morphine vs. vehicle). MCC950 treatment reduced spinal Caspase-1 activity in morphine-treated PAE female mice that displayed allodynia reversal (* p = 0.002). (B) In males, Caspase-1 activity was elevated between allodynic morphine-treated PAE mice and morphine-treated sac mice (p = 0.004, unpaired *t*-test). MCC950 treatment reduced spinal Caspase-1 activity in morphine-treated PAE male mice that displayed allodynia reversal (p = 0.04, unpaired *t*-test). (C) Despite all mice being exposed to minor nerve injury, morphine treatment significantly increased IL-1β protein levels in PAE female and male mice compared to sac mice ($ p < 0.005, PAE vs. Sac, morphine-treated mice) and PAE mice without morphine treatment (## p < 0.003, PAE morphine vs. PAE vehicle). MCC950 treatment reduced spinal IL-1β protein in morphine-treated PAE mice that displayed allodynia reversal (* p < 0.05). We observed significant sex differences in IL-1β protein, with females exhibiting higher levels compared to males in allodynic minor nerve-injured morphine-treated PAE mice (# p = 0.0001).

**Fig. 5.**
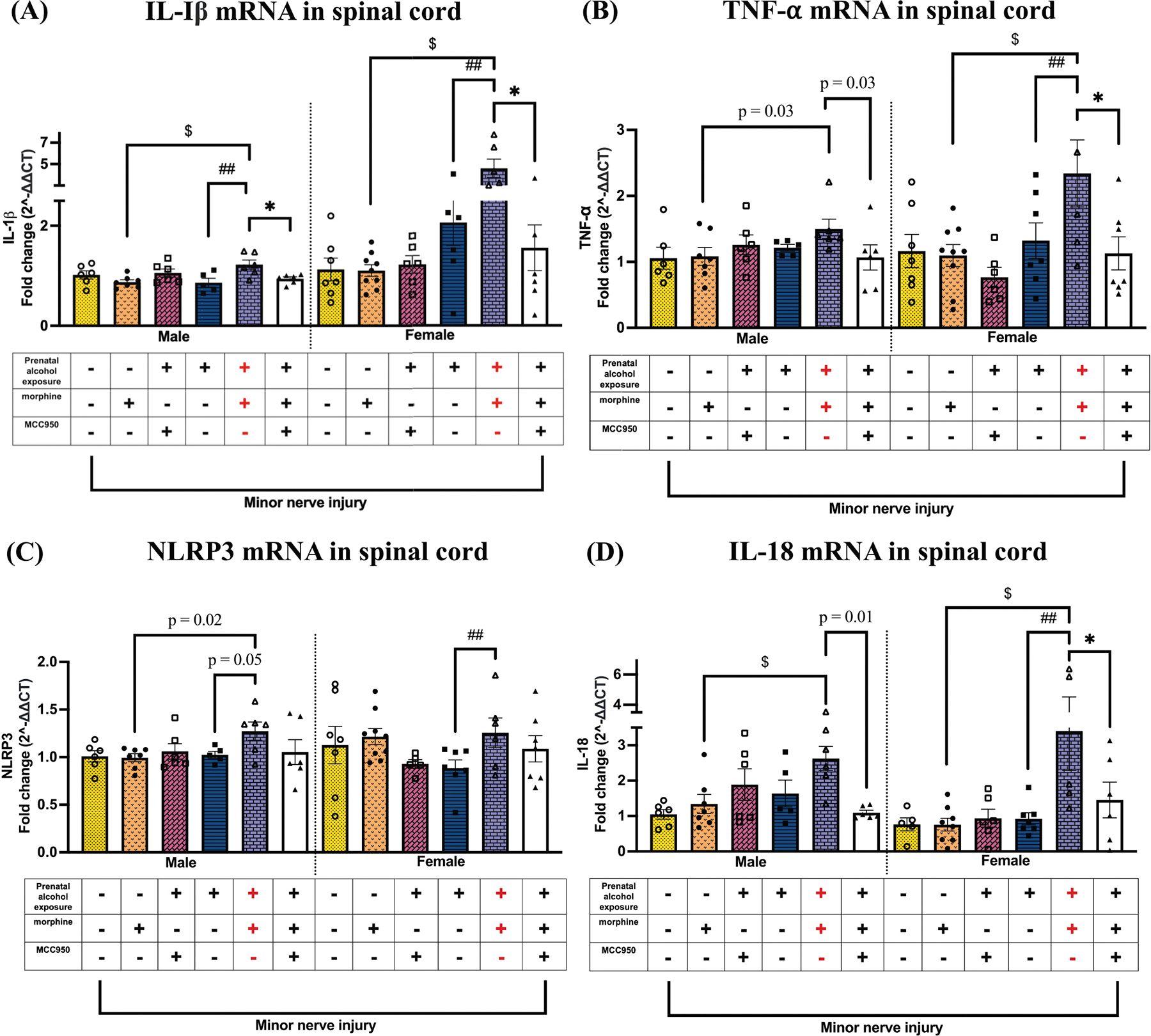
Effects of PAE, morphine interactions, and MCC950 treatment on IL-1β, NLRP3, and TNF-α mRNA levels in the spinal cord from minor-nerve injured mice. Spinal cords were collected from behaviorally verified mice, as represented in Fig. 2. Prenatal exposure (+), morphine (+), and MCC950 (−) in purple denote the allodynic group; all other groups are non-allodynic or allodynia-reversed at this post-CCI time point. (A) Despite all mice being exposed to minor nerve injury, morphine treatment significantly increased IL-1β mRNA in both sexes in PAE mice compared to sac mice ($p < 0.0008, PAE vs. sac, morphine-treated mice) and PAE mice without morphine treatment (## p < 0.03, PAE, morphine vs. vehicle). Regardless of sex, MCC950 treatment reduced IL-1β mRNA in morphine-treated PAE mice that displayed allodynia reversal (* p < 0.004). (B) In males, morphine-prolonged allodynia coincided with a significant increase in TNF-α mRNA in PAE mice compared to sac mice (p = 0.03, unpaired *t*-test, PAE vs. sac, morphine-treated mice). In females, morphine-prolonged allodynia coincided with a significant increase in TNF-α mRNA in PAE mice compared to sac mice ($ p = 0.0005, PAE vs. sac, morphine-treated mice) and PAE mice without morphine treatment (## p = 0.006, PAE, morphine vs. vehicle). Regardless of sex, MCC950 treatment significantly reduced TNF-α mRNA levels in morphine-treated PAE mice that displayed allodynia reversal (female: * p = 0.009; male: p = 0.03, unpaired *t*-test). (C) In males, morphine-prolonged allodynia coincided with a significant increase in NLRP3 mRNA in PAE mice compared to sac mice (p = 0.02, unpaired *t*-test, PAE vs. sac, morphine-treated mice) and PAE mice without morphine treatment (p = 0.05, unpaired *t*-test, morphine vs. vehicle). In females, regardless of minor nerve injury, morphine treatment significantly increased NLRP3 mRNA levels in morphine-treated PAE mice compared to vehicle-treated PAE mice (## p = 0.004). (D) In males and females, regardless of minor nerve injury, morphine treatment significantly increased IL-18 mRNA levels in PAE mice compared to morphine-treated sac male mice ($ p < 0.03) and vehicle-treated PAE mice (female: ## p = 0.0002; male: p = 0.01, unpaired *t*-test). Regardless of sex, MCC950 treatment significantly reduced IL-18 mRNA levels in morphine-treated PAE mice that displayed allodynia reversal (* p = 0.03).

**Fig. 6.**
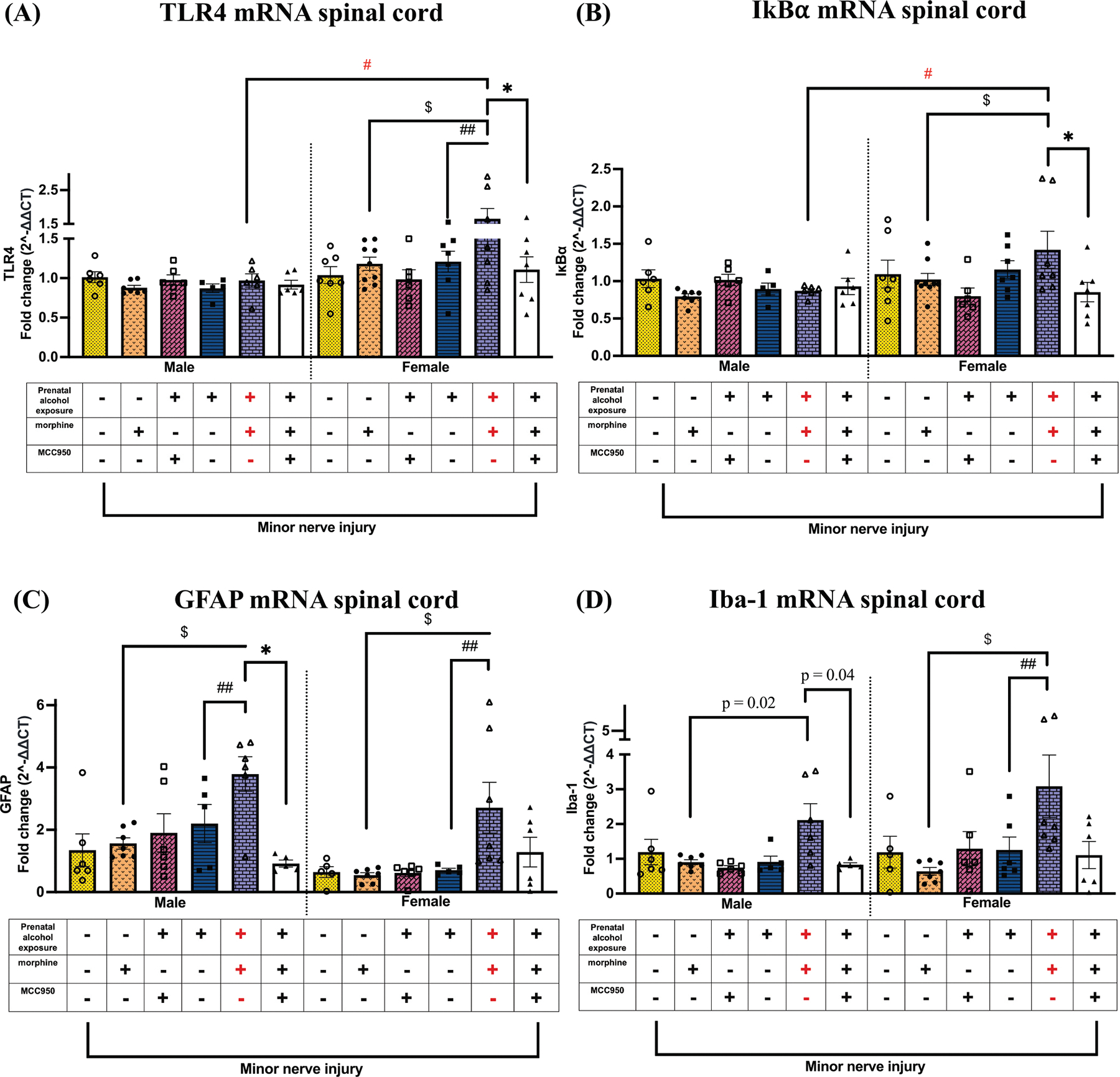
Effects of PAE, morphine and MCC950 treatment on TLR4 and IκBα mRNA levels in the spinal cord. (A) Morphine treatment increased TLR4 mRNA in the spinal cord only in allodynic PAE females compared to sac mice ($ p = 0.01, PAE vs. sac, morphine-treated mice) as well as vehicle-treated PAE mice (## p = 0.03). MCC950 treatment reduced TLR4 mRNA in the spinal cord in female PAE mice that displayed allodynia reversal (* p = 0.05). No changes were observed in males. We observed significant sex differences in TLR4 mRNA, with females exhibiting higher levels compared to males in allodynic, minor nerve-injured, morphine-treated PAE mice (# p = 0.002). (B) Morphine treatment increased IκBα mRNA in the spinal cord only in allodynic PAE females compared to sac mice ($ p = 0.03, PAE vs. sac, morphine-treated mice). MCC950 treatment reduced IκBα mRNA in the spinal cord in morphine-treated female PAE mice that displayed allodynia reversal (* p = 0.02). IκBα mRNA levels were significantly higher in allodynic female, minor nerve-injured, morphine-treated PAE mice than in their male counterparts (# p = 0.006). (C) Despite all mice being exposed to minor nerve injury, morphine treatment significantly increased GFAP mRNA in the spinal cord of allodynic PAE mice compared to sac mice regardless of sex ($ p < 0.04, PAE vs. sac, morphine-treated mice) and PAE mice without morphine treatment (## p < 0.03, PAE, morphine vs. vehicle). In males, MCC950 treatment reduced GFAP mRNA in morphine-treated male PAE mice that displayed allodynia reversal (* p = 0.005). (D) Despite all mice being exposed to minor nerve injury, morphine treatment significantly increased Iba-1 mRNA in the spinal cord of allodynic PAE mice compared to sac mice regardless of sex (female: $ p < 0.04; male: p = 0.02, unpaired *t*-test, PAE vs. sac, morphine-treated mice) and PAE mice without morphine treatment (## p < 0.03, PAE, morphine vs. vehicle). In males, MCC950 treatment reduced Iba-1 mRNA in morphine-treated male PAE mice that displayed allodynia reversal (p = 0.04, unpaired *t*-test).

**Fig. 7.**
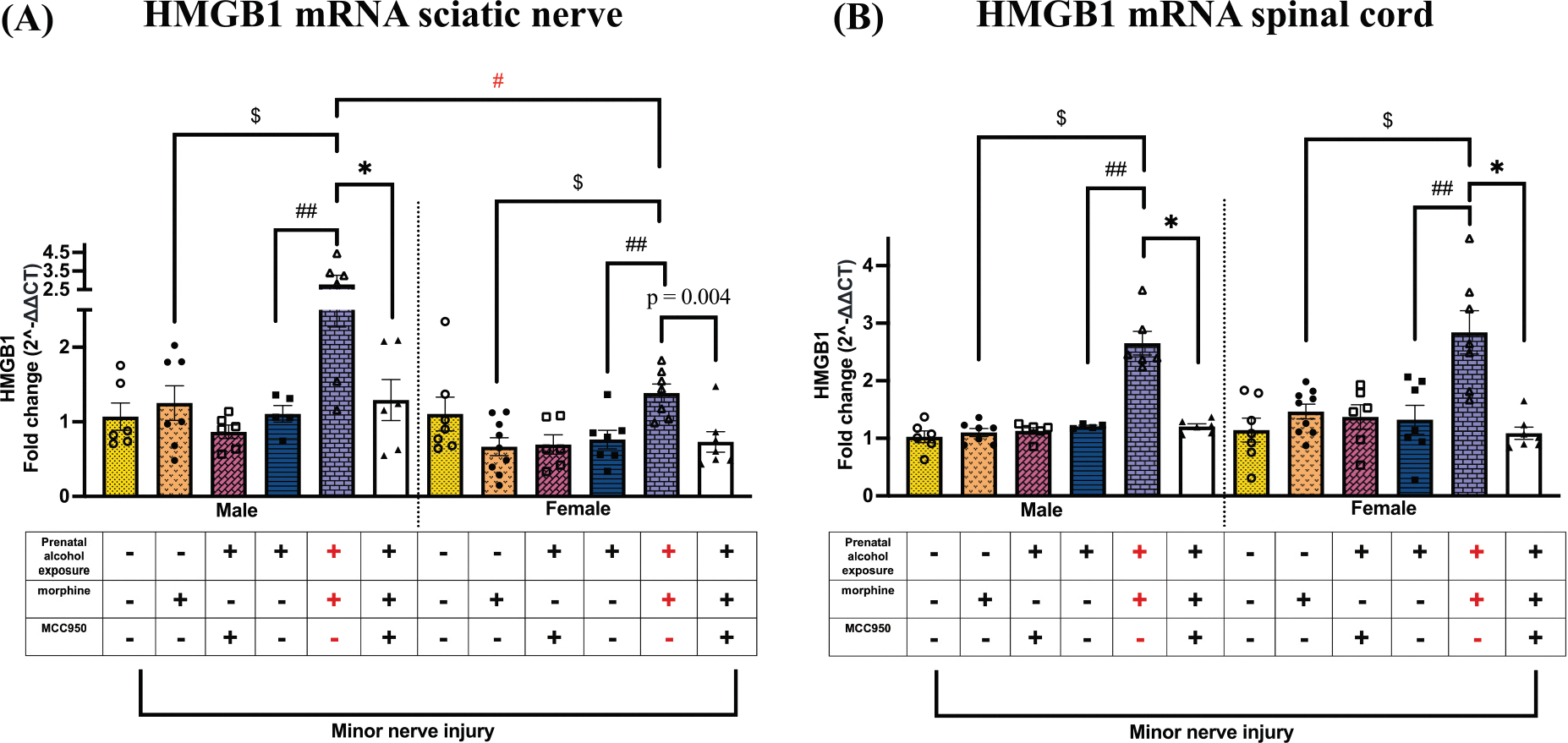
Effects of PAE and morphine and MCC950 treatment on HMGB1 mRNA levels in the injured nerve and spinal cord. (A) Despite all mice being exposed to minor nerve injury, and in both sexes, morphine treatment significantly increased HMGB1 mRNA in the sciatic nerve of allodynic PAE mice compared to sac mice ($ p < 0.03, PAE vs. sac, morphine-treated mice) and PAE mice without morphine treatment (## p < 0.03, PAE, morphine vs. vehicle). MCC950 treatment reduced HMGB1 mRNA in morphine-treated male PAE mice that displayed allodynia reversal (male: * p < 0.002; female: p = 0.004, unpaired *t*-test). We observed that HMGB1 mRNA was significantly higher in allodynic male, minor nerve-injured, morphine-treated PAE mice compared to their female counterparts (# p = 0.001). (B) Independent of minor nerve injury, morphine treatment significantly increased spinal HMGB1 mRNA in both sexes in PAE morphine-treated mice compared to sac morphine-treated mice ($ p < 0.0001, PAE vs. sac, morphine-treated mice) and PAE mice without morphine treatment (## p < 0.0003, PAE, morphine vs. vehicle). MCC950 treatment reduced HMGB1 mRNA in allodynia-reversed mice (* p < 0.0004).

**Fig. 8.**
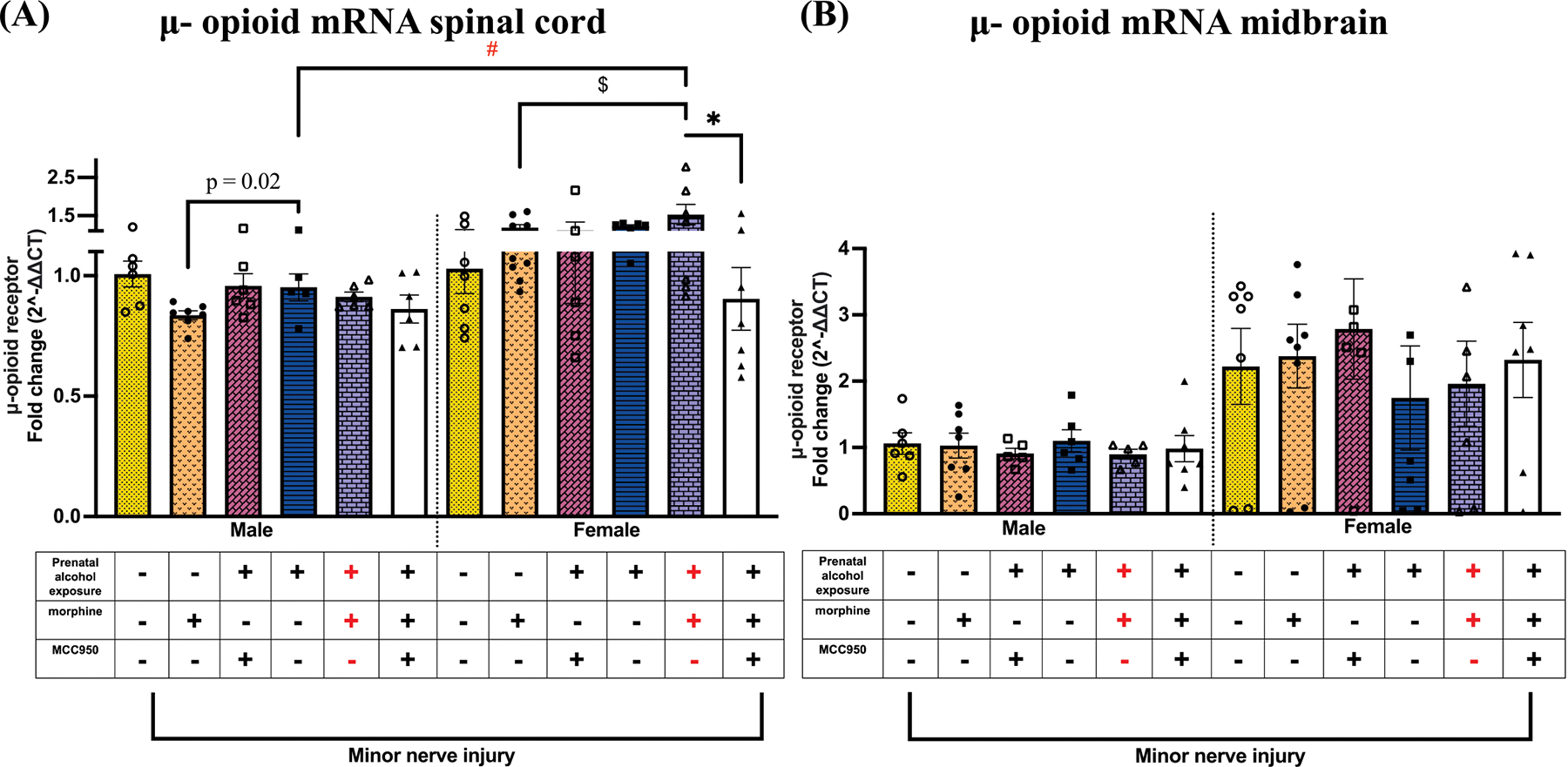
Effects of PAE and morphine immune interactions and MCC950 treatment on μ-opioid receptor mRNA in the spinal cord and midbrain in minor-nerve injured mice. (A) Regardless of sex, morphine-treated PAE mice exhibited significantly higher μ-opioid receptor mRNA levels compared to morphine-treated saccharin controls (female: $ p = 0.03; male: p = 0.02, unpaired *t*-test). μ-Opioid receptor mRNA was significantly higher in allodynic female, minor nerve-injured, morphine-treated PAE mice compared to their male counterparts (# p = 0.0007). MCC950 treatment reduced HMGB1 mRNA in allodynia-reversed mice (* p < 0.01) (B) No change was observed in the midbrain.

**Fig. 9.**
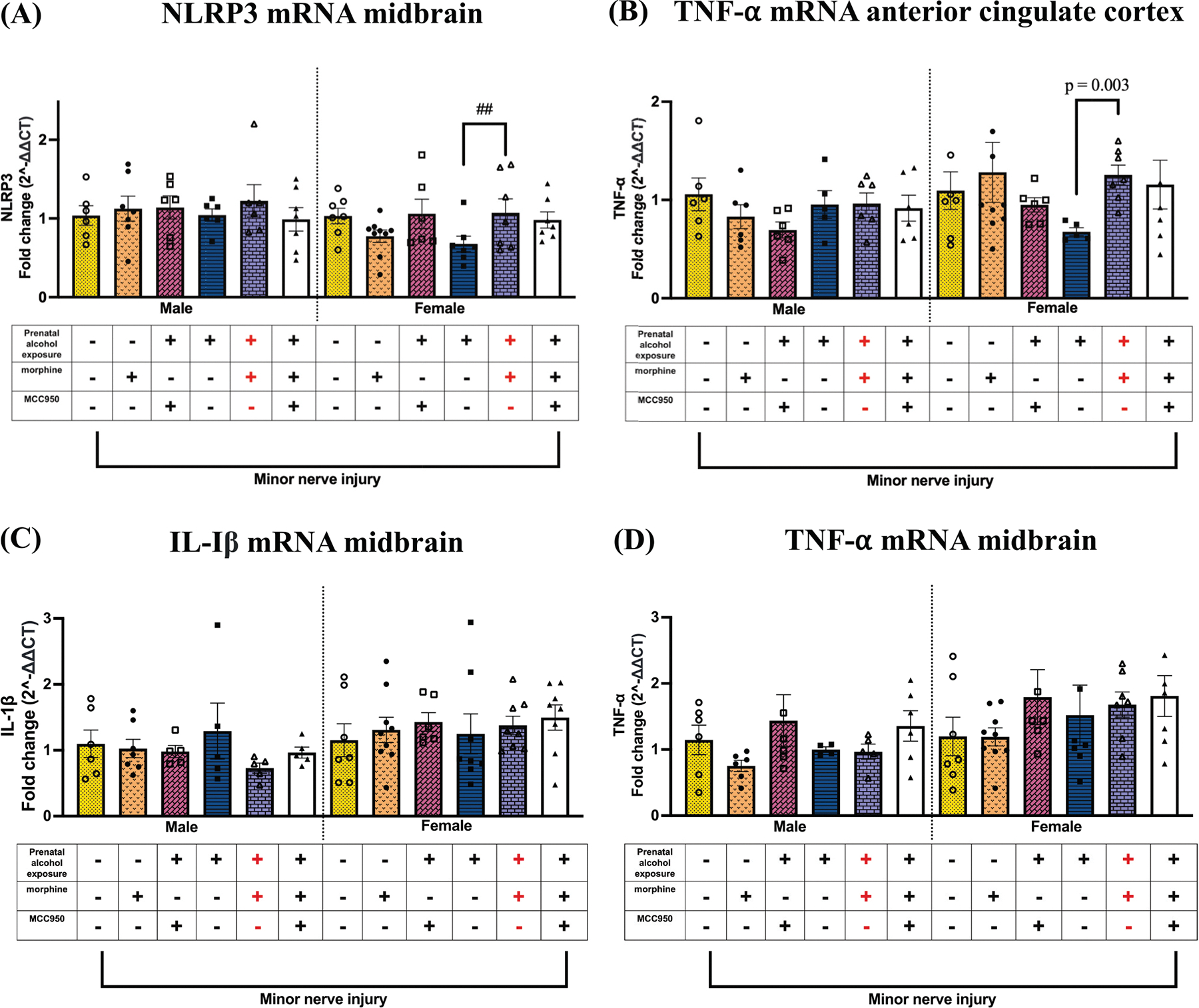
Effects of PAE and morphine and MCC950 on NLRP3 and TNF-α mRNA in the midbrain and anterior cingulate cortex. (A) Morphine treatment significantly increased NLRP3 mRNA in the midbrain of female PAE mice treated with morphine compared to PAE mice without morphine treatment (## p = 0.02). (B) Morphine treatment significantly increased TNF-α mRNA in the anterior cingulate cortex of female PAE mice treated with morphine compared to PAE mice without morphine treatment (p = 0.003, unpaired *t*-test). (C & D) No changes were observed in IL-1β or TNF-α mRNA in the midbrain regardless of sex.

Based on the clinical relevance and strong scientific premise from prior work, we focused on mechanical allodynia in this study, while other pain modalities such as mechanical or thermal hyperalgesia, may also be influenced via PAE and morphine interaction and differentially regulated by MCC950 (Chen et al., 2021), which warrants further investigation. Moreover, to avoid confounding factors from multiple behavioral assessments, we have not assessed these mice for other behavioral effects such as anxiety, locomotor activities (Hodgson et al., 2010; Ozdemir et al., 2024) that may be influenced by PAE and morphine. A total of 153 adult mice, including sac (control) and PAE offspring, were used in hind paw sensitivity assessments reported in this study. To explore the effect of morphine during minor nerve injury, adult mice with prenatal alcohol exposure (PAE) and age-matched non-PAE (sac) mice underwent sham injury or minor CCI and received morphine or vehicle treatment. Hind paw sensitivity was assessed at various time intervals to track the progression of allodynia until mice exhibited hind paw sensitivity comparable to baseline levels (Fig. 1). For Fig. 1, a total of 58 mice, 29 male, and 29 female mice, were used, with an N = 4–5 per treatment group. To specifically assess the role of NLRP3 in the maintenance of morphine-prolonged allodynia beyond the period of spontaneous recovery (typically resolved by day 21 in PAE mice, shown by our previous work ^38^) MCC950 or vehicle treatment were administered on day 23, when persistent allodynia could be attributed to neuroimmune dysregulation rather than nerve injury alone. (Fig. 2). For Fig. 2, a total of 95 mice, 46 male, and 49 female mice, were used, 6–11 mice per treatment group. Based on our previous molecular comparisons (effect size, *f* = 0.5) (Noor et al., 2020), an a priori power analysis (G*Power, α = 0.05, power = 0.80, 8 groups) indicated a total sample size of 34 mice (~6/group) to detect differences between allodynic and non-allodynic mice. Tissues were collected from all 95 mice, and molecular changes were assessed in each sample. Data are presented in Figs. 3–9 and all Supplemental Figures. Two samples were excluded from the molecular analysis due to notes indicating potential tissue compromise during tissue collection and processing.

### 2.13. Statistical analysis

#### 2.13.1. Behavioral data analysis

All behavioral data were analyzed using GraphPad Prism (GraphPad Software Inc.; RRID: SCR_002798) or RStudio. For Figs. 1 and 2, baseline comparisons were performed by collapsing groups across surgery, morphine, and MCC950 conditions, and a two-way ANOVA (sex × PAE) was used to assess group differences. To evaluate the development of allodynia after surgery, groups were collapsed by surgery condition (minor nerve injury vs. sham), and a three-way repeated measures (RM) ANOVA (sex × PAE × surgery × time) was conducted. Tukey’s post hoc tests were used to assess differences between PAE + minor nerve injury, sac + minor nerve injury, and PAE + sham groups. For Fig. 2A, sham mice were not part of the design; therefore, a two-way RM ANOVA (sex × PAE × time) was used. Post-morphine time points were analyzed separately using a three-way RM ANOVA (sex × PAE × morphine × time), with Tukey’s test applied to assess whether PAE + minor nerve injury + morphine mice exhibited greater allodynia compared to sac + minor nerve injury + morphine and PAE + minor nerve injury + vehicle mice. Sham groups were excluded from this analysis. Potential sex differences in morphine response were also evaluated. The effects of MCC950 were analyzed using a two-way RM ANOVA (sex × MCC950 × time), with Tukey’s test applied to compare PAE + minor nerve injury + morphine + vehicle and PAE + minor nerve injury + morphine + MCC950 groups.

#### 2.13.2. Molecular data analysis

Normality was assessed using the Shapiro-Wilk test. To maintain consistency across behavioral and molecular analyses, the same group variables and post hoc strategies were applied unless otherwise specified in the figure legends. Molecular outcomes related to morphine-prolonged allodynia were analyzed using a three-way ANOVA (sex × PAE × morphine), with Fisher’s LSD test used for post hoc comparisons. Sex differences in molecular responses were also evaluated. Molecular outcomes related to MCC950 effects were analyzed using a two-way ANOVA (sex × MCC950), with Fisher’s LSD post hoc tests. Adjusted *p*-values are reported, and all data are presented as mean ± SEM. In cases where the respective ANOVA did not yield significant values on post hoc comparisons from primary groups of interest unpaired t-tests was used based on our *a priori* hypothesis. Outliers were identified using Grubbs’ Test (GraphPad QuickCalc Outlier Calculator; α = 0.05). In figures depicting molecular data, Y-axes were broken to improve visualization across groups with widely varying expression levels. Main effects are reported in Table 1.

## 3. Results

### 3.1. Repeated treatment of moderate doses of morphine results in the prolongation of minor nerve injury-induced allodynia, only in PAE mice

A well-established model of minor chronic constriction injury was utilized to examine the potential interaction of PAE and morphine. Adult PAE mice and non-PAE control (sac) mice were exposed to minor CCI or sham surgery. Prior to surgery, PAE and sac control mice displayed similar levels of baseline (BL) hind paw sensitivity. Following sham surgery, their hind paw sensitivities remained similar to pre-surgery BL values (Fig. 1). Replicating our prior reports (Sanchez et al., 2017); minor CCI resulted in chronic unilateral allodynia only in PAE mice, whereas minor nerve-injured sac mice did not develop allodynia. A main effect of PAE (F_1,30_ = 33.54, p < 0.0001) was observed on hind paw responses ipsilateral to the sciatic damaged side from D1–D14 post-CCI time points; no sensitivity was detected in the contralateral side (Fig. 1A–B). At D14 post-CCI, mice were treated with a moderate dose of morphine for five consecutive days. Mice were tested every 2–4 days until all mice displayed ipsilateral hind paw sensitivities similar to their BL values. Comparing hind paw responses at post-morphine time points in nerve-injured mice, a main effect of PAE (F_1,30_ = 200.75, p < 0.0001) was observed. In both male and female sac mice, morphine treatment did not increase ipsilateral or contralateral paw sensitivity. In contrast, minor nerve-injured male PAE mice continued to display unilateral allodynia until D47 for males and D34 for females post-CCI following morphine treatment, whereas vehicle-treated nerve-injured PAE mice spontaneously reversed by D19 (Fig. 1A, C). A significant interaction effect of PAE × morphine (F_1,29_ = 182.39, p < 0.0001) was observed, with notable differences between allodynic morphine-treated PAE mice and vehicle-treated PAE mice from D19 to D34 in females and from D19 to D47 in males. In both male and female mice, no changes in hind paw sensitivity were observed in the contralateral hind paw at post-morphine treatment time points; nerve-injured PAE mice remained at about BL through the entire time course (Fig. 1B, D). There was a significant sex × time interaction (F_6,180_ = 3.39, p = 0.0034), suggesting that males and females exhibit different trajectories of morphine allodynia development over time. A significant sex × PAE × time interaction was also observed (F_6,180_ = 2.90, p = 0.0102).

### 3.2. NLRP3 inflammasome is necessary for the development of morphine-induced prolonged allodynia in nerve-injured PAE mice

Utilizing the small molecule inhibitor MCC950, we explored the key role of NLRP3 inflammasome activation in driving morphine-induced allodynia under PAE conditions. Following the same experimental paradigm described in Fig. 1, minor nerve-injured sac or PAE mice underwent morphine or vehicle treatment, followed by MCC950 administration. Reproducing the observations in Fig. 1, both female and male minor nerve-injured PAE mice developed robust allodynia, whereas sac + minor CCI mice remained stably non-allodynic. A main effect of PAE was observed only on the ipsilateral side of the injury (F_1,364_ = 11.17, p < 0.0009; Fig. 2A, C). In both sexes, morphine treatment further prolonged ongoing allodynia only in nerve-injured PAE mice (Fig. 2A, C). Analyzing post-morphine treatment time points, a significant PAE × morphine interaction was observed (F_1,86_ = 132.77, p < 0.0001). Following the development of morphine-induced allodynia in PAE mice, a single injection of MCC950 or vehicle was administered at day 23 post-CCI. In both sexes, post hoc comparisons revealed a significant difference between non-allodynic PAE + minor CCI + morphine + MCC950 mice and allodynic PAE + minor CCI + morphine + vehicle mice at 90 min post-MCC950 injection, with allodynia reversal persisting at 24 h (day 24; # p < 0.01 for both time points; Fig. 2A, C). These data confirm the critical role of NLRP3 inflammasome activation in sustaining morphine-prolonged allodynia in PAE mice, regardless of sex.

### 3.3. Morphine-induced prolonged allodynia in PAE mice is associated with elevated NLRP3 activity, as indicated by increased Caspase-1 levels and other downstream pro-inflammatory molecules near the injured nerve, which are reduced by MCC950 treatment

Following behavioral assessments (Fig. 2), tissues were collected, and the injured sciatic nerves were analyzed. Key molecular comparisons included: morphine-treated control (non-allodynic) vs. PAE (allodynic) mice, vehicle-treated (non-allodynic) PAE mice vs. allodynic PAE mice, and MCC950-treated pain-reversed PAE mice vs. allodynic PAE mice. Notably, at day 24 post-CCI, despite all mice being exposed to minor nerve injury, most treatment groups were non-allodynic due to spontaneous reversal or MCC950 treatment—except for the PAE + morphine + vehicle group, which remained allodynic (Fig. 2). Levels of Caspase-1 activity were measured at the injury site (Fig. 3A). In both males and females, significantly increased caspase-1 activity was observed in the allodynic group (PAE + morphine + vehicle) compared to non-allodynic controls: sac + morphine + vehicle ($ p <0.0001$) and PAE + vehicle + vehicle (## p < 0.0001). MCC950 treatment significantly reduced caspase-1 activity in morphine-treated PAE mice that displayed allodynia reversal (* p < 0.0001) in both sexes. Between-sex comparisons revealed significantly higher caspase-1 activity in males compared to females during morphine-prolonged allodynia (# p < 0.0001). In males and females, significantly increased levels of IL-1β protein (Fig. 3B) were observed in the allodynic group (PAE + morphine + vehicle) when compared to other non-allodynic control groups, sac + morphine + vehicle ($ p < 0.0009) and PAE + vehicle + vehicle (## p < 0.004). In both sexes, MCC950 treatment dramatically reduced IL-1β protein in morphine-treated PAE mice that displayed allodynia reversal (* p <0.04). Sciatic nerve mRNA levels of IL-1β, NLRP3, and TNF-α were also evaluated (Fig. 3C–E). In females, morphine-induced allodynia in PAE mice was associated with higher levels of IL-1β mRNA (Fig. 3C) compared to the non-allodynic control groups ($ p < 0.0001). MCC950 treatment reduced IL-1β mRNA in morphine-treated PAE females (* p = 0.01). In females, there was a significant increase in TNF-α mRNA in allodynic nerve-injured morphine-treated PAE mice compared to vehicle-treated PAE mice (## p = 0.04). Similarly, in males, allodynic nerve-injured morphine-treated PAE mice displayed greater TNF-α mRNA levels compared to sac morphine-treated mice (p = 0.02) (Fig. 3D). Significant sex differences in TNF-α mRNA levels were observed, with females displaying greater fold increases compared to males in nerve-injured morphine-treated PAE mice (# p = 0.04). Interestingly, MCC950 treatment reduced TNF-α mRNA in morphine-treated male PAE mice that displayed allodynia reversal (* p = 0.03). In males, morphine-induced allodynia was also associated with higher NLRP3 mRNA levels (Fig. 3E) compared to non-allodynic controls (p < 0.05). NLRP3 mRNA levels were significantly higher in male PAE mice compared to females during morphine-mediated allodynia (# p < 0.0001). MCC950 treatment significantly decreased NLRP3 mRNA levels in males compared to morphine-treated PAE mice with persistent allodynia (* p = 0.007). No NLRP3 mRNA changes were observed in females across treatment groups.

### 3.4. Morphine-induced prolonged allodynia in PAE mice is associated with upregulated NLRP3-related proinflammatory molecules in the spinal cord, which are reduced by MCC950 treatment

Ipsilateral lumbar spinal cord dorsal horn tissues were evaluated for molecular changes associated with TLR4–NLRP3 proinflammatory signaling. Caspase-1 activity and IL-1β protein levels were assessed to evaluate inflammasome activation in the spinal cord of both male and female mice. In males (Fig. 4B), Caspase-1 activity was elevated in PAE + morphine + vehicle mice compared to sac + morphine + vehicle controls (p = 0.004) and was reduced following MCC950 treatment (p = 0.04). In females (Fig. 4A), Caspase-1 activity was elevated in PAE + morphine + vehicle mice compared to PAE + vehicle + vehicle controls ($ p < 0.0001; ## p < 0.0001). MCC950 treatment significantly reduced spinal caspase-1 activity in morphine-treated PAE mice (* p = 0.002). For IL-1β protein (Fig. 4C), regardless of sex, morphine treatment significantly increased IL-1β levels in PAE mice compared to both sac + morphine + vehicle ($ p < 0.005) and PAE + vehicle + vehicle (## p <0.003) controls. MCC950 treatment significantly reduced spinal IL-1β protein levels in morphine-treated PAE mice (* p < 0.05). Higher IL-1β protein levels were observed in female PAE mice compared to males during morphine-mediated allodynia (# p = 0.0001). A similar pattern was observed at the transcript level. IL-1β mRNA (Fig. 5A) was significantly increased in both male and female PAE + morphine + vehicle mice during morphine-prolonged allodynia, compared to sac + morphine + vehicle controls ($ p < 0.0008) and PAE + vehicle + vehicle (## p < 0.03). In both sexes, MCC950-mediated allodynia reversal was associated with downregulation of spinal IL-1β mRNA (* p < 0.004). Morphine-prolonged allodynia also resulted in significant increases in TNF-α mRNA levels (Fig. 5B) in both males and females compared to sac + morphine + vehicle controls ($ p < 0.04). In females, spinal TNF-α mRNA was significantly higher in PAE + morphine + vehicle mice compared to PAE + vehicle + vehicle (## p = 0.006). In both sexes, MCC950-mediated allodynia reversal was associated with a decrease in TNF-α mRNA levels (* p < 0.04). In males, NLRP3 mRNA levels (Fig. 5C) were significantly increased in the PAE + morphine + vehicle group compared to non-allodynic controls (sac + morphine + vehicle, $ p = 0.02; PAE + vehicle + vehicle, ## p = 0.05). In females, NLRP3 mRNA was also elevated in allodynic PAE + morphine + vehicle mice compared to PAE + vehicle + vehicle controls (## p = 0.004), and there was a trend toward significance when compared to sac + morphine–treated mice (p = 0.07). No significant changes in NLRP3 mRNA were observed following MCC950 treatment in either sex. Finally, morphine treatment significantly increased IL-18 mRNA levels (Fig. 5D) in the spinal cords of both male and female PAE mice compared to sac + morphine + vehicle controls ($ p < 0.03). In females, IL-18 mRNA was also significantly elevated compared to PAE + vehicle + vehicle mice (## p = 0.0002). MCC950 treatment significantly reduced IL-18 mRNA levels in both sexes, coinciding with behavioral reversal of allodynia (* p < 0.04).

### 3.5. Morphine-induced prolonged allodynia in female PAE mice is associated with upregulated TLR4 and IκBα expression in the spinal cord, which is reduced by MCC950 treatment

We explored potential changes in TLR4 and IκBα mRNA levels in the spinal cord and sciatic nerve. Interestingly, while no changes were observed in male mice, spinal TLR4 mRNA levels (Fig. 6A) were significantly increased in morphine-treated female PAE mice compared to non-allodynic sac + morphine + vehicle controls ($ p = 0.01) and PAE + vehicle + vehicle mice (## p = 0.03). MCC950 treatment reduced spinal TLR4 mRNA levels in females (* p = 0.05). Similarly, spinal IκBα mRNA (Fig. 6B) did not change in males regardless of PAE, morphine, or MCC950 treatment. However, in females, IκBα mRNA levels were significantly higher in allodynic PAE + morphine + vehicle mice compared to sac + morphine + vehicle controls ($ p = 0.03). MCC950 treatment significantly reduced IκBα mRNA in female PAE mice (* p = 0.02). A comparison between sexes revealed that during morphine-induced allodynia, female PAE mice exhibited significantly greater fold increases in both TLR4 and IκBα mRNA compared to males (# p < 0.02). No significant differences in TLR4 or IκBα mRNA levels were observed across groups or sexes in the sciatic nerve (Supplemental Fig. 1B, C). However, sciatic nerve TLR4 and IκBα mRNA fold changes were significantly higher in allodynic, minor nerve-injured, morphine-treated male PAE mice compared to their female counterparts (# p < 0.03).

### 3.6. Morphine-induced prolonged allodynia in PAE mice is associated with cell-specific increases in Iba-1 and GFAP expression in both sexes

Given the established roles of GFAP and Iba-1 as markers of astrocyte and microglial activation, respectively, we examined whether PAE and morphine interactions promote cell-specific neuroimmune changes associated with morphine-prolonged allodynia (Fig. 6C). Regardless of sex, morphine-prolonged allodynia in PAE mice was associated with higher levels of GFAP mRNA compared to sac + morphine + vehicle mice ($ p <0.04) and PAE + vehicle + vehicle controls (## p <0.03). In males, MCC950 treatment reduced GFAP mRNA, though not significantly (* p < 0.05). In females, morphine-prolonged allodynia significantly increased Iba-1 mRNA in PAE + morphine + vehicle mice compared to both sac + morphine + vehicle ($ p < 0.05) and PAE + vehicle + vehicle controls (## p < 0.007; Fig. 6D). In males, Iba-1 mRNA was significantly increased in morphine-treated PAE mice compared to sac + morphine + vehicle controls (p = 0.02). In males, MCC950-mediated allodynia reversal was associated with reduced Iba-1 mRNA levels (p = 0.04).

### 3.7. Upregulation of HMGB1 at the level of the injured nerve and spinal cord may contribute to morphine-induced prolonged allodynia in PAE mice, which is reduced following MCC950 treatment

Given evidence that spinal HMGB1 acts as an endogenous immune activator in morphine-induced hyperalgesia and allodynia under non-PAE conditions (Yang et al., 2021; Feldman et al., 2012), we investigated whether PAE and morphine interactions further disrupt HMGB1 levels, potentially contributing to morphine-prolonged allodynia. Our data suggest that in both sexes, minor nerve injury led to a dramatic increase in sciatic nerve HMGB1 mRNA levels in morphine-treated PAE mice compared to morphine-treated sac controls ($ p < 0.03) or vehicle-treated PAE mice (## p < 0.03; Fig. 7A). MCC950-mediated allodynia reversal was associated with reduced HMGB1 mRNA levels in both sexes (* p < 0.005); significance in females was determined by a *t*-test, while the effect in males emerged from a post hoc ANOVA comparison, indicating a more robust difference. Additionally, sciatic HMGB1 upregulation revealed a sex difference: male PAE mice exhibited more pronounced HMGB1 elevation than female PAE mice in the presence of morphine-prolonged allodynia (# p = 0.0001). In addition to the increased HMGB1 expression at the peripheral nerve injury site, spinal cord HMGB1 mRNA levels were also significantly upregulated (Fig. 7B) in both sexes compared to morphine-treated sac controls ($ p < 0.0001) and vehicle-treated PAE mice (## p < 0.0003). Notably, MCC950-mediated allodynia reversal was associated with a significant decrease in spinal HMGB1 mRNA levels (* p < 0.0004).

### 3.8. Female PAE mice displayed increased spinal μ-opioid receptor mRNA levels during morphine-prolonged allodynia, which is decreased by MCC950 treatment

μ-Opioid receptor (MOR) mRNA was analyzed to assess potential modulatory effects of PAE, morphine, and MCC950 treatment. In both male and female PAE mice, morphine treatment significantly increased spinal μ-opioid receptor mRNA levels compared to morphine-treated sac controls ($ p < 0.04; Fig. 8A). In females, MCC950 treatment significantly reduced μ-opioid receptor mRNA levels (* p < 0.01). Notably, morphine-treated female PAE mice exhibited significantly higher fold changes in μ-opioid receptor mRNA expression in the spinal cord compared to their male counterparts (# p = 0.0007). No significant changes were observed in midbrain μ-opioid receptor mRNA levels across groups (Fig. 8B).

### 3.9. Morphine-prolonged allodynia is associated with increased NLRP3 and TNF-α levels in different pain-relevant brain regions in females

**Fig. 10.**
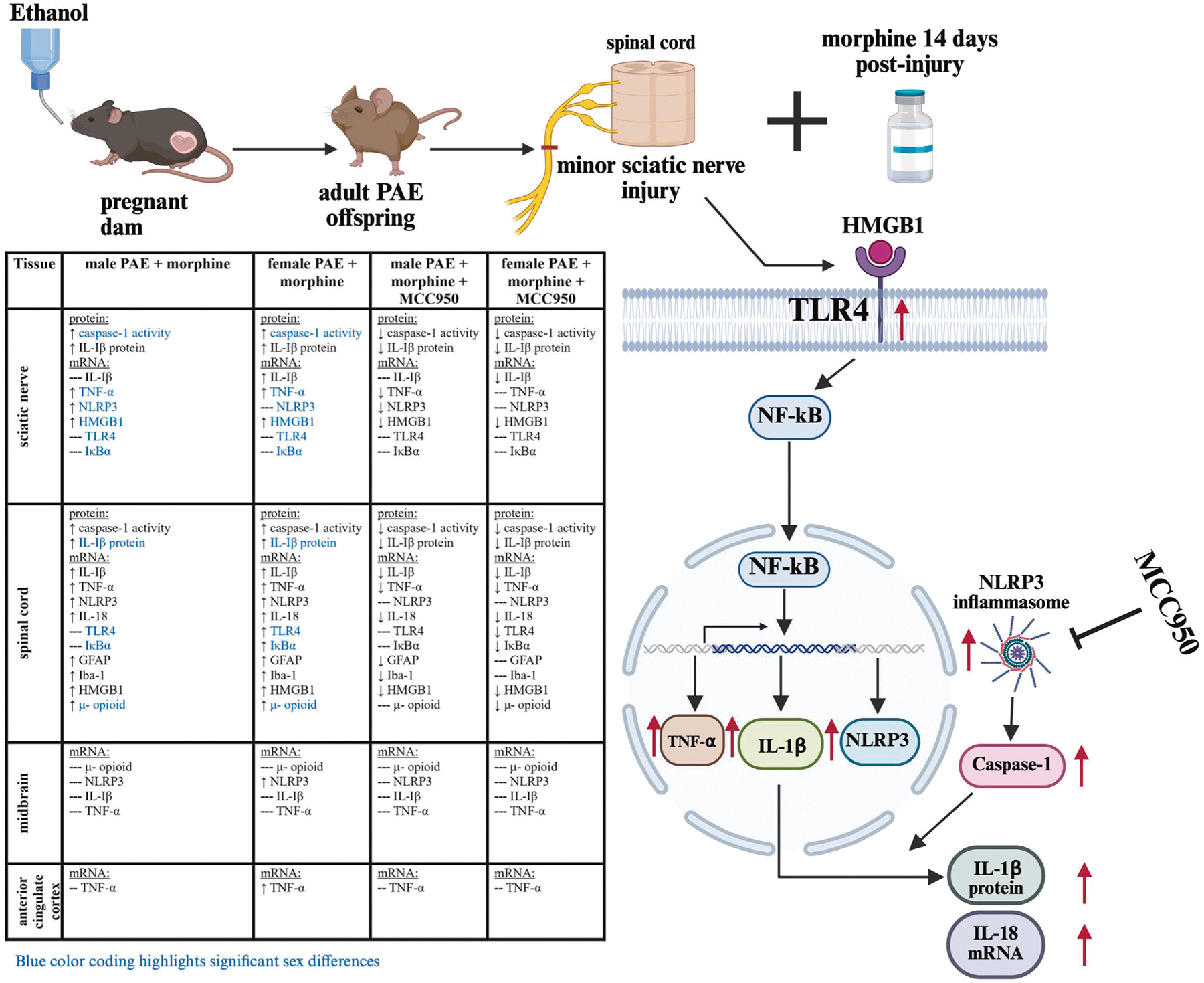
Schematic diagram of the experimental paradigm and summary of key findings of this study. This figure was created with Biorender.com.

To explore whether the interaction between PAE and morphine during nerve injury extends beyond the spinal cord and involves activation of TLR4 and NLRP3 signaling in pain-relevant brain regions, two key areas, the midbrain and anterior cingulate cortex (ACC), were analyzed. In females, elevated NLRP3 mRNA levels were detected in the midbrain of PAE mice with morphine-induced allodynia compared to vehicle-treated PAE controls (## p = 0.02), while NLRP3 expression remained unchanged in males (Fig. 9A). No significant changes were observed in IL-1β or TNF-α mRNA levels in the midbrain. In the ACC, allodynic PAE female mice exhibited significantly greater fold increases in TNF-α mRNA (Fig. 9B) compared to non-allodynic, minor nerve-injured, vehicle-treated PAE controls (# p = 0.003). No MCC950-mediated changes were observed in any of these molecular markers in either the midbrain or ACC. A summary of molecular findings across pain relevant regions is depicted in Fig. 10

## 4. Discussion

Preclinical and clinical studies suggest neuroimmune dysregulation is a primary driver of FASD-related pathophysiology (Sn et al., 2019; Sanchez et al., 2017; Noor et al., 2017; Bodnar et al., 2020). TLR4 signaling modulates both immediate and long-term effects of PAE (Pascual et al., 2017). Increased IL-1β has been reported due to alcohol exposure or PAE, following secondary immune activation (Sanchez et al., 2019). However, mechanistic insights confirming the necessary role of NLRP3 inflammasomes in PAE-induced CNS dysfunction are sparse. Our prior study demonstrated that systemic blockade of NLRP3, as well as blocking spinal IL-1β signaling (Sanchez et al., 2019), mitigates PAE-induced susceptibility to allodynia. Based on this strong scientific premise, we examined NLRP3 as a nexus of immune dysregulation consequent to PAE and opioid use in the context of nerve injury. While blocking NLRP3 activity with MCC950, which crosses the blood–brain barrier (Fan et al., 2018; Martínez-Martel et al., 2025), has been shown to reverse allodynia in female PAE mice (Noor et al., 2023), it remains unclear whether this prolonged allodynia results from peripheral and/or central immune signaling and how MCC950 exerts its beneficial effects. In line with our previous findings (Noor et al., 2023), this study generated novel behavioral and molecular data demonstrating that (1) PAE is a risk factor for morphine-induced allodynia in both male and female PAE mice, with a longer duration observed in males; (2) NLRP3 inflammasome activation is a key player in morphine and PAE neuroimmune interactions during nerve injury in both sexes; and (3) associated with upregulation of TLR4 and NLRP3-related factors in pain-relevant PNS and CNS regions, inhibiting NLRP3 activation reduces these critical proinflammatory factors, contributing to the reversal of allodynia.

### 4.1. PAE and morphine immune interaction during nerve injury drive NLRP3-dependent prolonged allodynia, regardless of sex

We find that morphine treatment significantly increases the chronicity of allodynia in minor nerve-injured PAE mice. Notably, morphine alone did not induce chronic allodynia in the absence of nerve injury, as evidenced by the lack of contralateral allodynia. Nonetheless, neither morphine nor PAE alone is sufficient to induce prolonged allodynia in the context of minor nerve injury. Instead, prolonged allodynia reflects immune sensitization arising from the interplay of these factors, unmasking PAE-induced susceptibility to morphine-mediated chronic pain outcomes. While our study examined the effects of morphine, similar effects may extend to other opioids, such as fentanyl and oxycodone, that activate TLR4 (Green-Fulgham et al., 2019; Hutchinson et al., 2010). Although unknown in the context of morphine-mediated allodynia, sex differences in allodynia (Osborne and Davis, 2016; Linher-Melville et al., 2020; Nicotra et al., 2014; Rosen et al., 2017) and PAE (Bake et al., 2023; Candelaria-Cook et al., 2024; Paudel et al., 2020) have been reported in prior studies, as well as morphine (Juni et al., 2008). Although we have observed a similar duration of minor nerve-induced allodynia in PAE mice without morphine treatment, these data are the first to suggest that the interaction between PAE and morphine results in a more extended duration of allodynia in males, compared to females.

### 4.2. PAE and morphine peripheral immune interaction involve heightened NLRP3 inflammasome activation at the site of injured nerve

Sciatic nerve injury causes localized nerve damage, which recruits heterogeneous immune cell populations, including macrophages, to the injury site (Relja and Land, 2020). These macrophages release proinflammatory cytokines and mediators (e.g., HMGB1) that contribute to peripheral sensitization and the enhancement of synaptic transmission in the dorsal horn of the spinal cord (Yang et al., 2021), facilitating the development and maintenance of central sensitization underlying chronic pain. Data presented in our study indicate that augmented peripheral immune activity with elevated Caspase-1 and other TLR4-related proinflammatory mediators engages in morphine-mediated allodynia under PAE conditions, consistent with primed activity of peripheral PAE macrophages (Noor et al., 2023). Moreover, MCC950 effectively reversed allodynia and reduced IL-1β and Caspase-1 levels, as well as mRNA levels of IL-1β and TNF-α. Although MCC950 acts at the level of “activation” of the NLRP3 inflammasome complex, which is downstream of NF-κB-mediated transcription of NLRP3 and IL-1β, TNF-α, these observations at 24 h post-MCC950 treatment are likely due to suppression of cytokine signaling, disrupting this feed-forward loop of NF-κB activation (Jiao et al., 2020; Zeng et al., 2021; Blevins et al., 2022; Chen et al., 2020).

### 4.3. PAE and morphine immune interaction upregulated spinal glial activation and NLRP3- and TLR4-related factors following minimal nerve injury

The critical role of spinal IL-1β signaling and activation of microglia and astrocytes is well-documented during allodynia (Li et al., 2022; Yuan et al., 2024). Our data revealed increased levels of key mediators of allodynia: Caspase-1, IL-1β, NLRP3, TNF-α and IL-18 (Chen et al., 2021), concurrent with spinal microglia and astrocyte activation in PAE mice with morphine-prolonged allodynia across both sexes (Noor et al., 2023; Fiore et al., 2022). Reversal of allodynia following MCC950 treatment was associated with a reduction of these proinflammatory factors (Jiao et al., 2020; Perera et al., 2018), as well as glial activation markers. Similar observations were made by prior studies showing NLRP3 inhibition attenuates GFAP mRNA and other neuroinflammatory factors during chronic pain states (Li et al., 2022; Yuan et al., 2024). MCC950 specifically blocks NLRP3 inflammasome (Coll et al., 2015), but not other inflammasomes (Coll et al., 2015; Matico et al., 2025) (such as NLRC4) that may contribute to Caspase-1 activity. Therefore, our data confirm the critical role of NLRP3-dependent Caspase-1 activity driving persistent allodynia in morphine-treated PAE mice, regardless of sex. Future studies examining the effects of NLRP3 inhibition at the spinal cord level could provide insights into whether ameliorating NLRP3 in both the PNS and/or CNS is necessary for allodynia reversal.

One critical aspect of this model to consider is that morphine is metabolized within a few hours to days (Murphy et al., 2025). Therefore, prolonged allodynia is likely to stem from morphine and PAE interaction that may enhance pain-promoting endogenous factors (Relja and Land, 2020). Morphine has been shown to directly activate immune cells via binding to MD2 (Wang et al., 2012) (an adaptor protein of TLR4) and may promote HMGB1 release, which occurs in both the peripheral immune or CNS glia, consistent with our prior findings on peripheral macrophages and the existing literature (Noor et al., 2023; Mo et al., 2023; Qian et al., 2020). At both the sciatic nerve and spinal cord levels, we have observed increased levels of HMGB1 in PAE mice, and the reversal of allodynia by MCC950 is associated with reduced HMGB1, suggesting that persistent allodynia in PAE mice may be attributed to ongoing TLR4 activation by HMGB1. These mRNA data are consistent with prior studies that support increased levels of HMGB1 protein and confirm its critical contribution in the context of repeated morphine exposure, resulting in chronic excitation of the TLR4-NF-κB axis (Ellis et al., 2016; Grace et al., 2016; Grace et al., 2018). Further mechanistic approaches are needed to determine whether HMGB1 plays a necessary role in sustaining morphine-prolonged allodynia in PAE mice. In addition to morphine-mediated actions on immune cells, indirect mechanisms of immune modulation may also be involved through interactions with the neuroendocrine system. Repeated exposure to morphine may blunt the hypothalamic–pituitary–adrenal (HPA) responsiveness and dysregulate immune function (Haroutounian, 2018; Houshyar et al., 2001). Separately, PAE disrupts the HPA axis during adulthood, with hyperactive HPA in response to stress and a blunted response to cortisol associated with heightened proinflammatory factors (Lecuyer et al., 2017; Pearson et al., 2015; Raineki et al., 2016; Ruffaner-Hanson et al., 2023). Lastly, chronic pain can act as a stressor (Abdallah and Geha, 2017; Aboushaar and Serrano, 2024; Wyns et al., 2023). Therefore, PAE and morphine interactions via the HPA axis are likely involved, which is a critical area to be explored in future studies.

### 4.4. Sex-specific responses during PAE and morphine-mediated immune interactions in the presence of nerve injury

During allodynia, variable spinal expression of NLRP3 and differential involvement of TLR4 has been reported (Bruno et al., 2018; Noor et al., 2017; Bettoni et al., 2008). The critical role of the spinal Caspase-1 has been confirmed during morphine-mediated allodynia in non-PAE male rats (Grace et al., 2016). This study is the first to conduct a side-by-side comparison of TLR4-NLRP3-related proinflammatory molecules, revealing sexual dimorphism in PAE and morphine-mediated immune interactions. To summarize our molecular data common to both sexes, we observed upregulation of Caspase-1 activity, HMGB1, and IL-1β in both the periphery and the spinal cord, along with spinal glial activation, in PAE mice with morphine-induced prolonged allodynia. Moreover, MCC950 treatment reversed allodynia and dampened these factors in both sexes. Despite the critical role of NLRP3 activation regardless of sex, our molecular data revealed distinct inflammatory profiles in the peripheral versus CNS across the sexes. We found that PAE females display a more robust spinal immune sensitization than males along the TLR4-NF-κB-IL-1β pathway; elevated levels of TLR4, IκB-α, IL-1β, TNF-α, and IL-18 were observed. Interestingly, in the periphery, despite similar levels of IL-1β protein in males and females and upregulation of IL-1β and TNF-α mRNA levels, changes in NLRP3 were observed only in males. This disconnect may reflect the contribution of other NF-κB-independent mechanisms (such as MAPK/AP-1) that may contribute to the mRNA transcription of these cytokines, which are upstream molecules of NLRP3 activation. Also, differential post-transcriptional control of the stability of these mRNAs may occur (Duez and Pourcet, 2021; Wan et al., 2022). In contrast, in the periphery, male PAE mice exhibited more pronounced TLR4-related inflammatory factors (Noor et al., 2019) with comparatively greater fold increases of TLR4 and IκB-α mRNA levels than in females, and with significant increases in NLRP3 mRNA and IL-1β protein. Typically, IκB-α transcription occurs due to NF-κB activity, providing negative feedback to its own activation (Ferreiro and Komives, 2010; Yan et al., 2020). While no changes in IκB-α mRNA may infer a lack of robust ongoing NF-κB activity at the injury site in PAE females at this time point, in a chronic inflammation scenario, IκB-α is often blunted and dysregulated, contributing to sustained inflammation (Hinz and Scheidereit, 2014; Yee et al., 2021). Therefore, more targeted approaches, such as nuclear localization of NF-κB or IκB-α activity, may pinpoint the involvement of NF-κB or other critical transcription factors that influence the transcriptional regulation of these factors. Moreover, TLR4-NF-κB-mediated transcription promotes NLRP3 actions and vice versa. However, transcriptional upregulation of all components of the inflammasome complex may not be required for NLRP3 actions; rather, their active transcription and stability of these transcripts could be temporally regulated during chronic inflammation and vary between sexes (Linher-Melville et al., 2020; Cowie et al., 2019; Silva Santos Ribeiro et al., 2023). Although observed only in the presence of PAE, increases in NLRP3 mRNA levels in the CNS appeared to be morphine-driven in females. Significant changes in NLRP3 transcripts were not observed in allodynic PAE vs non-PAE control mice, suggesting that NLRP3-inflammasome activity, rather than changes in NLRP3 mRNAs, is the primary driver of allodynia in female mice. Together, these data reflect potential differential effects of PAE and morphine on TLR4-NF-κB activity across sexes and in the PNS versus the CNS, yet converge on downstream NLRP3 inflammasome-mediated actions in both sexes.

Notably, spinal μ-opioid receptor mRNA expression was upregulated in PAE females during prolonged allodynia, in a similar pattern of spinal TLR4 and IκB-α changes, consistent with prior reports indicating the involvement of TLR4–NF–κB signaling in regulating the transcription of μ-opioid receptors (Gessi et al., 2016; Bettoni et al., 2008). Our initial characterization in pain-relevant brain regions (Kv et al., 2017; Kv et al., 2017) revealed that mRNA levels of most of these immune factors were unaltered. Interestingly, elevated mRNA levels of TNF-α in ACC, and NLRP3 in midbrain were detected in female PAE mice during morphine-prolonged allodynia. However, our dissection method captured PAG along with other adjacent midbrain nuclei and may not reflect subtle molecular alterations that may occur in males. Further investigation is required to gain a more comprehensive understanding of the role of NLRP3 within the pain-relevant brain regions.

Lastly, although we have conducted the molecular analysis at a time point when both male and female mice were stably allodynic, in light of our data suggesting a longer duration of allodynia in male PAE mice, greater magnitudes of spinal proinflammatory molecules were not evident in males. However, during morphine-prolonged allodynia, greater HMGB1 levels were observed in PAE males than in females at the sciatic nerve. HMGB1 may promote NLRP3 actions and allodynia via other TLR receptors as well as non-TLR pathways, leading to various other proinflammatory mediators (Das et al., 2016), which may work synergistically in PAE males. These data may also indicate potential differential regulation of pain resolution mechanisms that may involve dysregulation of anti-inflammatory molecules (Dengler et al., 2014; Je et al., 2023).

## 5. Limitations

Although our data validated protein levels of a few critical effector molecules, such as Caspase-1 and IL-1β, due to the insufficient tissue material, we prioritized mRNA profiling of other factors, employing a targeted approach to the signaling pathway of interest. Overall, our data convincingly support our conclusion, confirming the critical role of NLRP3 inflammasome activation during morphine-prolonged allodynia. Our mRNA findings provide evidence of transcriptional changes in various upstream components of the TLR4-NLRP3 pathway. However, the mRNA levels may not accurately reflect the protein levels of all these molecules. Additionally, our molecular detection of IL-1β protein could detect both pro- and mature IL-1β levels. Although this could introduce minor ambiguity in interpretation, excess unprocessed pro-IL-1β undergoes rapid degradation (Vijayaraj et al., 2021); hence, ELISA detection is commonly used to infer mature IL-1β levels. Moreover, our data revealed a similar pattern of IL-1β protein and caspase-1 activity, further reflecting changes in mature IL-1β in our samples. Our data also suggested potential involvement of IL-18. To conclude the necessary roles of IL-1β and/or IL-18, studies designed to block the functional effects of these pain-inducing cytokines during morphine-prolonged allodynia would be required. Lastly, this study provides a glimpse into the activation of spinal astrocytes and microglia in this context; however, potential contributions of different glial cell types remain unclear. Although upregulation of these glial activation markers is often indicative of pro-inflammatory activation, the data presented here should be interpreted with caution, as these findings are insufficient to distinguish their pro- and anti-inflammatory phenotype and their contributions to adverse chronic pain outcomes from PAE and morphine.

## 6. Conclusions

We conclude that PAE poses a risk factor for peripheral and central immune sensitization from later-life exposure to morphine as a chronic pain therapeutic. NLRP3 inflammasome activation is a central driver of worsened allodynia outcome in PAE conditions. Targeting NLRP3 may be beneficial in mitigating neuroimmune dysfunction and adverse effects of morphine, as well as neurobehavioral deficits associated with FASD.

## Supplementary Material

MMC1

## Figures and Tables

**Table 1:** Key molecular findings: main effects.

Tissue	Target	Main effects

**Sciatic nerve**	Caspase-1 activity	PAE: F_1, 20_ = 83.85, p < 0.001 morphine: F_1, 25_ = 85.76, p < 0.001 PAE x morphine: F_1, 20_ = 53.35, p < 0.001 sex x PAE x morphine: F_1, 20_ = 25.75, p < 0.001 MCC950: F_1, 22_ = 105.3, p < 0.001
	IL-Iβ protein	morphine: F_1, 45_ = 11.5, p = 0.002 sex (within morphine treatment): F_1, 45_ = 17.16, p = 0.0001 morphine x sex: F_1, 45_ = 0.4, p = 0.002 MCC950: F_1, 21_ = 18.29, p = 0.0003
	IL-Iβ mRNA	PAE: F_1, 21_ = 12.30, p = 0.002 morphine: F_1, 25_ = 13.21, p = 0.001 PAE x morphine: F_1, 21_ = 10.25, p = 0.004 MCC950: F_1, 22_ = 9.55, p = 0.005
	NLRP3 mRNA	PAE: F_1, 21_ = 8.51, p = 0.008 sex (within morphine treatment): F_1, 25_ = 14.24, p = 0.0009 PAE x sex: F_1, 21_ = 11.47, p = 0.003 MCC950: F_1, 22_ = 5.06, p = 0.03
	TNF-α mRNA	PAE: F_1, 45_ = 17.05, p = 0.002 morphine: F_1, 45_ = 8.81, p = 0.005 sex (within morphine treatment): F_1, 45_ = 5.37, p = 0.03 MCC950: F_1, 22_ = 6.58, p = 0.02
	HMGB1 mRNA	PAE: F_1, 21_ = 17.64, p = 0.0004 morphine: F_1, 25_ = 6.66, p = 0.02 sex (within morphine treatment): F_1, 25_ = 8.55, p = 0.007 PAE x morphine: F_1, 21_ = 23.95, p < 0.001 MCC950: F_1, 22_ = 14.39, p = 0.001
	TLR4 mRNA	sex (within morphine treatment): F_1, 25_ = 116, p = 0.002 PAE x sex: F_1, 21_ = 5.32, p = 0.03
	IkBα mRNA	sex (within morphine treatment): F_1, 25_ = 10.7, p = 0.003 PAE x sex: F_1, 21_ = 6.83, p = 0.02
**Spinal cord**	Caspase-1 activity female	PAE: F_1, 20_ = 8.91, p = 0.008 morphine: F_1, 20_ = 13.13, p = 0.002 PAE x morphine: F_1, 20_ = 13.08, p = 0.002
	IL-Iβ protein	PAE: F_1, 13_ = 310, p < 0.0001 morphine: F_1, 13_ = 17.85, p = 0.001 sex (within morphine treatment): F_1, 9_ = 40.03, p = 0.0001 Morphine x sex: F_1, 9_ = 24.28, p = 0.0008 PAE x sex: F_1, 9_ = 16.53, p = 0.003 MCC950: F_1, 13_ = 19.28, p = 0.0007
	IL-Iβ mRNA	PAE: F_1, 19_ = 15.99, p = 0.0008 morphine: F_1, 25_ = 38.98, p < 0.001 PAE x morphine: F_1, 19_ = 8.16, p = 0.01 MCC950: F_1, 20_ = 25.50, p < 0.001
	TNF-α mRNA	morphine: F_1, 45_ = 7.99, p = 0.007 MCC950: F_1, 21_ = 7.29, p = 0.01
	NLRP3 mRNA	PAE: F_1, 21_ = 4.86, p = 0.04
	IL-I8 mRNA	PAE: F_1, 17_ = 13.27, p = 0.002 morphine: F_1, 24_ = 15.38, p = 0.0006 PAE x morphine: F_1, 17_ = 9.27, p = 0.007 MCC950: F_1, 19_ = 9.07, p = 0.007
	TLR4 mRNA	sex (within morphine treatment): F_1, 46_ = 11.35, p = 0.002
	IkBα mRNA	sex (within morphine treatment): F_1, 46_ = 10.70, p = 0.002
	HMGB1 mRNA	PAE: F_1, 45_ = 30.08, p < 0.0001 morphine: F_1, 45_ = 28.83, p < 0.0001 PAE x morphine: F_1, 45_ = 17.57, p = 0.0001 MCC950: F_1, 22_ = 47.26, p < 0.0001
	μ-opioid mRNA	sex (within morphine treatment): F_1, 25_ = 12.34, p = 0.002 PAE x sex: F_1, 20_ = 4.59, p = 0.04
	GFAP mRNA	PAE: F_1, 39_ = 10.35, p = 0.003 morphine: F_1, 39_ = 19.68, p < 0.0001 sex (within morphine treatment): F_1, 39_ = 8.44, p = 0.006 PAE x morphine: F_1, 39_ = 5.2, p = 0.03 MCC950: F_1, 21_ = 11.04, p = 0.003
	Ibal mRNA	morphine: F_1, 41_ = 6.78, p = 0.01 PAE x morphine: F_1, 41_ = 8.597, p = 0.006
**Midbrain**	μ-opioid mRNA	sex (within morphine treatment): F_1, 45_ = 8.001, p = 0.007 PAE x sex: F_1, 20_ = 4.59, p = 0.04
	NLRP3 mRNA	PAE x morphine: F_1, 21_ = 6.211, p = 0.02
	TNF-α mRNA	sex (within morphine treatment): F_1, 23_ = 5.16, p = 0.03

## Data Availability

Data will be made available on request.

## Appendix A. Supplementary data

Supplementary data to this article can be found online at https://doi.org/10.1016/j.bbi.2025.06.041.

## References

[R1] Ab C, Tf C-C, Mj S, 2020. Unisensory and multisensory responses in fetal alcohol spectrum disorders (FASD): effects of spatial congruence. Neuroscience 430. 10.1016/j.neuroscience.2020.01.013 PMC7098157 31982473

[R2] Abdallah CG, Geha P, 2017. Chronic pain and chronic stress: two sides of the same coin? Chronic Stress (thousand Oaks) 1. 10.1177/2470547017704763 PMC5546756 28795169

[R3] Aboushaar N, Serrano N, 2024. The mutually reinforcing dynamics between pain and stress: mechanisms, impacts and management strategies. Front. Pain Res. 5, 1445280. 10.3389/fpain.2024.1445280 PMC11609167 39624230

[R4] Alboni S, Cervia D, Sugama S, Conti B, 2010. Interleukin 18 in the CNS. J. Neuroinflamm. 7, 9. 10.1186/1742-2094-7-9 PMC2830964 20113500

[R5] Al-Hasani R, Bruchas MR, 2011. Molecular mechanisms of opioid receptor-dependent signaling and behavior. Anesthesiology 115, 1363–1381. 22020140 10.1097/ALN.0b013e318238bba6 PMC3698859

[R6] Bake S, Rouzer SK, Mavuri S, Miranda RC, Mahnke AH, 2023. The interaction of genetic sex and prenatal alcohol exposure on health across the lifespan. Front. Neuroendocrinol. 71, 101103. 37802472 10.1016/j.yfrne.2023.101103 PMC10922031

[R7] Barreiro-Iglesias A, Shifman MI, 2012. Use of fluorochrome-labeled inhibitors of caspases to detect neuronal apoptosis in the whole-mounted lamprey brain after spinal cord injury. Enzyme Res. 10.1155/2012/835731 PMC3399409 22829997

[R8] Begum E, Mahmod MR, Rahman MM, , 2024. IL-18 blockage reduces neuroinflammation and promotes functional recovery in a mouse model of spinal cord injury. Biomolecules 15. 10.3390/biom15010016 PMC11761924 39858411

[R9] Berta T, Liu T, Liu Y-C, Xu Z-Z, Ji R-R, 2012. Acute morphine activates satellite glial cells and up-regulates IL-1β in dorsal root ganglia in mice via matrix metalloprotease-9. Mol. Pain 8, 1744–8069–8–18. 10.1186/1744-8069-8-18 PMC3352126 22439811

[R10] Bettoni I, , 2008. Glial TLR4 receptor as new target to treat neuropathic pain: efficacy of a new receptor antagonist in a model of peripheral nerve injury in mice. Glia 56, 1312–1319. 18615568 10.1002/glia.20699

[R11] Bhatt M, Sharma M, Das B, 2024. The role of inflammatory cascade and reactive astrogliosis in glial scar formation post-spinal cord injury. Cell. Mol. Neurobiol. 44, 78. 39579235 10.1007/s10571-024-01519-9 PMC11585509

[R12] Blevins HM, Xu Y, Biby S, Zhang S, 2022. The NLRP3 inflammasome pathway: a review of mechanisms and inhibitors for the treatment of inflammatory diseases. Front. Aging Neurosci. 14, 879021. 35754962 10.3389/fnagi.2022.879021 PMC9226403

[R13] Bodnar TS, , 2020. Immune network dysregulation associated with child neurodevelopmental delay: modulatory role of prenatal alcohol exposure. J. Neuroinflamm. 17, 39. 10.1186/s12974-020-1717-8 PMC6988366 31992316

[R14] Breuer L, Greenmyer JR, Wilson T, 2024. Clinical diagnosis and management of fetal alcohol spectrum disorder and sensory processing disorder in children. Children 11. 10.3390/children11010108 PMC10814837 38255421

[R15] Bruno K, , 2018. Targeting toll-like receptor-4 (TLR4)-an emerging therapeutic target for persistent pain states. Pain 159, 1908–1915. 29889119 10.1097/j.pain.0000000000001306 PMC7890571

[R16] Candelaria-Cook FT, , 2024. Sex-specific differences in Resting Oscillatory Dynamics in Children with Prenatal Alcohol Exposure. Neuroscience 543, 121–136. 38387734 10.1016/j.neuroscience.2024.02.016 PMC10954390

[R17] Chen S-P, , 2019. Pharmacological inhibition of the NLRP3 inflammasome as a potential target for cancer-induced bone pain. Pharmacol. Res. 147, 104339. 31276771 10.1016/j.phrs.2019.104339

[R18] Chen F, , 2020. Melatonin alleviates intervertebral disc degeneration by disrupting the IL-1β/NF-κB-NLRP3 inflammasome positive feedback loop. Bone Res. 8, 10. 32133213 10.1038/s41413-020-0087-2 PMC7028926

[R19] Chen W, , 2022. The upregulation of NLRP3 inflammasome in dorsal root ganglion by ten-eleven translocation methylcytosine dioxygenase 2 (TET2) contributed to diabetic neuropathic pain in mice. J. Neuroinflammation 19, 302. 36527131 10.1186/s12974-022-02669-7 PMC9756585

[R20] Chen C, Smith MT, 2023. The NLRP3 inflammasome: role in the pathobiology of chronic pain. Inflammopharmacology 31, 1589–1603. 37106238 10.1007/s10787-023-01235-8 PMC10352419

[R21] Chen R, Yin C, Fang J, Liu B, 2021. The NLRP3 inflammasome: an emerging therapeutic target for chronic pain. J. Neuroinflamm. 18, 84. 10.1186/s12974-021-02131-0 PMC8008529 33785039

[R22] Coll RC, , 2015. A small-molecule inhibitor of the NLRP3 inflammasome for the treatment of inflammatory diseases. Nat. Med. 21, 248–255. 25686105 10.1038/nm.3806 PMC4392179

[R23] Cowie AM, Dittel BN, Stucky CL, 2019. A novel sex-dependent target for the treatment of postoperative pain: the NLRP3 inflammasome. Front. Neurol. 10, 622. 31244767 10.3389/fneur.2019.00622 PMC6581722

[R24] Cui C-X, , 2023. Research progress on the mechanism of chronic neuropathic pain. IBRO Neurosci Rep 14, 80–85. 36632243 10.1016/j.ibneur.2022.12.007 PMC9827377

[R25] Das N, Dewan V, Grace PM, , 2016. HMGB1 activates proinflammatory signaling via TLR5 leading to allodynia. Cell Rep. 17. 10.1016/j.celrep.2016.09.076 PMC5087801 27760316

[R26] Dengler EC, , 2014. Improvement of spinal non-viral IL-10 gene delivery by D-mannose as a transgene adjuvant to control chronic neuropathic pain. J. Neuroinflammation 11, 92. 24884664 10.1186/1742-2094-11-92 PMC4046049

[R27] Duez H, Pourcet B, 2021. Nuclear receptors in the control of the NLRP3 inflammasome pathway. Front. Endocrinol. 12, 630536. 10.3389/fendo.2021.630536 PMC7947301 33716981

[R28] Ellis A, Grace PM, Wieseler J, , 2016. Morphine amplifies mechanical allodynia via TLR4 in a rat model of spinal cord injury. Brain Behav. Immun. 58. 10.1016/j.bbi.2016.08.004 PMC5067205 27519154

[R29] Fan Y, , 2018. Inhibiting the NLRP3 Inflammasome with MCC950 Ameliorates Isoflurane-Induced Pyroptosis and Cognitive Impairment in Aged mice. Front. Cell. Neurosci. 12, 426. 30524241 10.3389/fncel.2018.00426 PMC6262296

[R30] Farsi D, Ghaffarzad A, Foroughinia R, Abdar S, Nouri M, 2013. A comparative study of 0.10 mg/kg vs 0.15 mg/kg intravenous morphine in acute limb trauma pain management. Ulus. Travma Acil Cerrahi Derg. 19, 398–404. 24214779 10.5505/tjtes.2013.86383

[R31] Feldman P, Due MR, Ripsch MS, Khanna R, White FA, 2012. The persistent release of HMGB1 contributes to tactile hyperalgesia in a rodent model of neuropathic pain. J. Neuroinflammation 9, 180. 22824385 10.1186/1742-2094-9-180 PMC3488576

[R32] Ferreiro DU, Komives EA, 2010. Molecular mechanisms of system control of NF-kappaB signaling by IkappaBalpha. Biochemistry 49, 1560–1567. 20055496 10.1021/bi901948j PMC2865148

[R33] Fiore NT, , 2022. Sex-specific transcriptome of spinal microglia in neuropathic pain due to peripheral nerve injury. Glia 70, 675–696. 35050555 10.1002/glia.24133 PMC8852349

[R34] Fjeldsted B, Xue L, 2019. Sensory processing in young children with fetal alcohol spectrum disorder. Phys. Occup. Ther. Pediatr. 39. 10.1080/01942638.2019.1573775 30947610

[R35] Gades NM, Danneman PJ, Wixson SK, Tolley EA, 2000. The influence of analgesic choice, administration, and duration on postoperative pain in rodents. Contemp. Top. Lab. Anim. Sci. 39, 8–13. 11487232

[R36] Gessi S, , 2016. The activation of μ-opioid receptor potentiates LPS-induced NF-kB promoting an inflammatory phenotype in microglia. FEBS Lett. 590, 2813–2826. 27427408 10.1002/1873-3468.12313

[R37] Grace PM, , 2018. Protraction of neuropathic pain by morphine is mediated by spinal damage associated molecular patterns (DAMPs) in male rats. Brain Behav. Immun. 72, 45–50. 28860068 10.1016/j.bbi.2017.08.018 PMC5832500

[R38] Grace PM, Strand KA, Galer EL, , 2016. Morphine paradoxically prolongs neuropathic pain in rats by amplifying spinal NLRP3 inflammasome activation. Proc. Natl. Acad. Sci. U. S. A. 113. 10.1073/pnas.1602070113 PMC4914184 27247388

[R39] Green-Fulgham SM, Ball JB, Kwilasz AJ, , 2019. Oxycodone, fentanyl, and morphine amplify established neuropathic pain in male rats. Pain 160. 10.1097/j.pain.0000000000001652 PMC7053537 31299018

[R40] Guo M, Jiang Z, Chen Y, Wang F, Wang Z, 2021. Inflammatory cytokines in midbrain periaqueductal gray contribute to diabetic induced pain hypersensitivity through phosphoinositide 3-kinase/protein kinase B/mammalian target of rapamycin signaling pathway. Korean J. Pain 34. 10.3344/kjp.2021.34.2.176 PMC8019962 33785669

[R41] Gwak YS, Hulsebosch CE, Leem JW, 2017. Neuronal-glial interactions maintain chronic neuropathic pain after spinal cord injury. Neural Plast. 2017, 2480689. 28951789 10.1155/2017/2480689 PMC5603132

[R42] Haroutounian S, 2018. Postoperative opioids, endocrine changes, and immunosuppression. PAIN Rep. 3, e640. 29756086 10.1097/PR9.0000000000000640 PMC5902248

[R43] He Y, Hara H, Núñez G, 2016. Mechanism and regulation of NLRP3 inflammasome activation. Trends Biochem. Sci. 41. 10.1016/j.tibs.2016.09.002 PMC5123939 27669650

[R44] He W, Long T, Pan Q, , 2019. Microglial NLRP3 inflammasome activation mediates IL-1β release and contributes to central sensitization in a recurrent nitroglycerin-induced migraine model. J. Neuroinflamm. 16. 10.1186/s12974-019-1459-7 PMC6456991 30971286

[R45] Hinz M, Scheidereit C, 2014. The IκB kinase complex in NF-κB regulation and beyond. EMBO Rep. 15, 46–61. 24375677 10.1002/embr.201337983 PMC4303448

[R46] Hodgson SR, Hofford RS, Roberts KW, Wellman PJ, Eitan S, 2010. Socially induced morphine pseudosensitization in adolescent mice. Behav. Pharmacol. 21, 112–120. 20215964 10.1097/FBP.0b013e328337be25 PMC2946112

[R47] Holdridge SV, Armstrong SA, Taylor AM, Cahill CM, 2007. Behavioural and morphological evidence for the involvement of glial cell activation in delta opioid receptor function: implications for the development of opioid tolerance. Mol. Pain 3, 1744–8069–3–7. 10.1186/1744-8069-3-7 PMC1828713 17352824

[R48] Houshyar H, Galigniana MD, Pratt WB, Woods JH, 2001. Differential responsivity of the hypothalamic-pituitary-adrenal axis to glucocorticoid negative-feedback and corticotropin releasing hormone in rats undergoing morphine withdrawal: possible mechanisms involved in facilitated and attenuated stress responses. J. Neuroendocrinol. 13, 875–886. 11679056 10.1046/j.1365-2826.2001.00714.x

[R49] Hutchinson MR, , 2007. Opioid-Induced glial activation: mechanisms of activation and implications for opioid analgesia, dependence, and reward. Sci. World J. 7, 746941. 10.1100/tsw.2007.230 PMC5901235 17982582

[R50] Hutchinson MR, , 2010. Evidence that opioids may have toll-like receptor 4 and MD-2 effects. Brain Behav. Immun. 24, 83–95. 19679181 10.1016/j.bbi.2009.08.004 PMC2788078

[R51] Je S, , 2023. The FDA-approved compound, pramipexole and the clinical-stage investigational drug, dexpramipexole, reverse chronic allodynia from sciatic nerve damage in mice, and alter IL-1β and IL-10 expression from immune cell culture. Neurosci. Lett. 814. 10.1016/j.neulet.2023.137419 PMC10552878 37558176

[R52] Jiao J, Zhao G, Wang Y, Ren P, Wu M, 2020. MCC950, a selective inhibitor of NLRP3 inflammasome, reduces the inflammatory response and improves neurological outcomes in mice model of spinal cord injury. Front. Mol. Biosci. 7, 37. 32195267 10.3389/fmolb.2020.00037 PMC7062868

[R53] Ju J, , 2024. Interleukin-18 in chronic pain: Focus on pathogenic mechanisms and potential therapeutic targets. Pharmacol. Res. 201, 107089. 38295914 10.1016/j.phrs.2024.107089

[R54] Juni A, Klein G, Kowalczyk B, 2008. Sex differences in hyperalgesia during morphine infusion: effect of gonadectomy and estrogen treatment. Neuropharmacology 54. 10.1016/j.neuropharm.2008.04.004 18457849

[R55] Ko F, Hh S, ME, Tsk P, 2019. The midbrain periaqueductal gray as an integrative and interoceptive neural structure for breathing. Neurosci. Biobehav. Rev. 98. 10.1016/j.neubiorev.2018.12.020 30611797

[R56] Kv S, Sl P, Lc F, 2017. Neuropathic pain-induced enhancement of spontaneous and pain-evoked neuronal activity in the periaqueductal gray that is attenuated by gabapentin. Pain 158. 10.1097/j.pain.0000000000000905 PMC5474198 28328571

[R57] Lecuyer M, , 2017. PLGF, a placental marker of fetal brain defects after in utero alcohol exposure. Acta Neuropathol. Commun. 5, 44. 28587682 10.1186/s40478-017-0444-6 PMC5461764

[R58] Li S, , 2022. Microglial NLRP3 inflammasome activates neurotoxic astrocytes in depression-like mice. Cell Rep. 41. 10.1016/j.celrep.2022.111532 36288697

[R59] Li H, Guan Y, Liang B, , 2022. Therapeutic potential of MCC950, a specific inhibitor of NLRP3 inflammasome. Eur. J. Pharmacol. 928. 10.1016/j.ejphar.2022.175091 35714692

[R60] Linher-Melville K, Shah A, Singh G, 2020. Sex differences in neuro(auto)immunity and chronic sciatic nerve pain. Biol. Sex Differ. 11, 62. 33183347 10.1186/s13293-020-00339-y PMC7661171

[R61] Livak KJ, Schmittgen TD, 2001. Analysis of relative gene expression data using real-time quantitative PCR and the 2(−Delta Delta C(T)) Method. Methods 25, 402–408. 11846609 10.1006/meth.2001.1262

[R62] Lm B, , 2013. Moderate prenatal alcohol exposure reduces plasticity and alters nmda receptor subunit composition in the dentate gyrus. J. Neurosci. 33. 10.1523/JNEUROSCI.1217-12.2013 PMC3563269 23325244

[R63] Lm B, Ma A, Kk C, 2012. A Limited access mouse model of prenatal alcohol exposure that produces long-lasting deficits in hippocampal-dependent learning and memory. Alcoholism 36. 10.1111/j.1530-0277.2011.01644.x PMC3572784 21933200

[R64] Ls T, Jm S, 2016. Effects of moderate prenatal alcohol exposure during early gestation in rats on inflammation across the maternal-fetal-immune interface and later-life immune function in the offspring. J. Neuroimmune Pharmacol. 11. 10.1007/s11481-016-9691-8 PMC5525056 27318824

[R65] Martínez-Martel I, Negrini-Ferrari SE, Pol O, 2025. MCC950 reduces the anxiodepressive-like behaviors and memory deficits related to paclitaxel-induced peripheral neuropathy in mice. Antioxidants (basel) 14. 10.3390/antiox14020143 PMC11851537 40002330

[R66] Matico R, , 2025. Navigating from cellular phenotypic screen to clinical candidate: selective targeting of the NLRP3 inflammasome. EMBO Mol. Med. 17, 54–84. 39653810 10.1038/s44321-024-00181-4 PMC11730736

[R67] Matsuoka T, Yoshimatsu G, Sakata N, , 2020. Inhibition of NLRP3 inflammasome by MCC950 improves the metabolic outcome of islet transplantation by suppressing IL-1β and islet cellular death. Sci. Rep. 10. 10.1038/s41598-020-74786-3 PMC7578017 33087823

[R68] Milligan ED, , 2000. Thermal hyperalgesia and mechanical allodynia produced by intrathecal administration of the human immunodeficiency virus-1 (HIV-1) envelope glycoprotein gp120. Brain Res. 861, 105–116. 10751570 10.1016/s0006-8993(00)02050-3

[R69] Milligan ED, Maier SF, Watkins LR, 2003. Review: neuronal-glial interactions in central sensitization. Semin. Pain Med. 1, 171–183.

[R70] Mo J, , 2023. PAG neuronal NMDARs activation mediated morphine-induced hyperalgesia by HMGB1-TLR4 dependent microglial inflammation. J. Psychiatr. Res. 164, 150–161. 37352811 10.1016/j.jpsychires.2023.05.082

[R71] Murphy PB, Patel P & Barrett MJ Morphine. in StatPearls (StatPearls Publishing, Treasure Island (FL), 2025). 30252371

[R72] Napadow V, Sclocco R, Henderson LA, 2019. Brainstem neuroimaging of nociception and pain circuitries. PAIN Rep. 4. 10.1097/PR9.0000000000000745 PMC6727990 31579846

[R73] Nicotra L, Tuke J, Grace PM, Rolan PE, Hutchinson MR, 2014. Sex differences in mechanical allodynia: how can it be preclinically quantified and analyzed? Front. Behav. Neurosci. 8. 10.3389/fnbeh.2014.00040 PMC3923156 24592221

[R74] Noor S, Milligan ED, 2018. Lifelong Impacts of moderate prenatal alcohol exposure on neuroimmune function. Front. Immunol. 9. 10.3389/fimmu.2018.01107 PMC5992426 29910801

[R75] Noor S, Sanchez JJ, Vanderwall AG, , 2017. Prenatal alcohol exposure potentiates chronic neuropathic pain, spinal glial and immune cell activation and alters sciatic nerve and DRG cytokine levels. Brain Behav. Immun. 61. 10.1016/j.bbi.2016.12.016 PMC5316367 28011263

[R76] Noor S, Sun MS, Vanderwall AG, Havard MA, Sanchez JE, Harris NW, Nysus MV, Norenberg JP, West HT, Wagner CR, Jantzie LL, Mellios N, Milligan ED, 2019. LFA-1 antagonist (BIRT377) similarly reverses peripheral neuropathic pain in male and female mice with underlying sex divergent peripheral immune proinflammatory phenotypes. Neuroimmunol. Neuroinflamm. 6, 10. 31763376 10.20517/2347-8659.2019.18 PMC6873931

[R77] Noor S, Sanchez JJ, Sun MS, , 2020. The LFA-1 antagonist BIRT377 reverses neuropathic pain in prenatal alcohol-exposed female rats via actions on peripheral and central neuroimmune function in discrete pain-relevant tissue regions. Brain Behav. Immun. 87. 10.1016/j.bbi.2020.01.002 PMC7316595 31918004

[R78] Noor S, Sun MS, Pasmay AA, , 2023. Prenatal alcohol exposure promotes NLRP3 inflammasome-dependent immune actions following morphine treatment and paradoxically prolongs nerve injury-induced pathological pain in female mice. Alcohol: Clin. Exp. Res. 47. 10.1111/acer.15214 PMC10764094 38151779

[R79] Ohashi Y, Uchida K, Fukushima K, Inoue G, Takaso M, 2023. Mechanisms of Peripheral and central sensitization in osteoarthritis pain. Cureus 15. 10.7759/cureus.35331 PMC9949992 36846635

[R80] Osborne NR, Davis KD, 2016. Sex differences in pain. Pain 157.

[R81] Ozdemir D, Meyer J, Kieffer BL, Darcq E, 2024. Model of negative affect induced by withdrawal from acute and chronic morphine administration in male mice. Sci. Rep. 14, 9767. 38684914 10.1038/s41598-024-60759-3 PMC11059349

[R82] Pascual M, , 2017. TLR4 response mediates ethanol-induced neurodevelopment alterations in a model of fetal alcohol spectrum disorders. J. Neuroinflamm. 14, 145. 10.1186/s12974-017-0918-2 PMC5525270 28738878

[R83] Paudel P, Pierotti C, Lozano E, , 2020. Prenatal alcohol exposure results in sex-specific alterations in circular RNA expression in the developing mouse brain. Front. Neurosci. 14. 10.3389/fnins.2020.581895 PMC7693437 33304235

[R84] Pearson J, Tarabulsy GM, Bussières E-L, 2015. Foetal programming and cortisol secretion in early childhood: a meta-analysis of different programming variables. Infant Behav. Dev. 40, 204–215. 26209745 10.1016/j.infbeh.2015.04.004

[R85] Perera AP, , 2018. MCC950, a specific small molecule inhibitor of NLRP3 inflammasome attenuates colonic inflammation in spontaneous colitis mice. Sci. Rep. 8, 8618. 29872077 10.1038/s41598-018-26775-w PMC5988655

[R86] Popova S, Dozet D, Shield K, Rehm J, Burd L, 2021. Alcohol’s Impact on the Fetus. Nutrients 13. 10.3390/nu13103452 PMC8541151 34684453

[R87] Qian J, , 2020. Chronic morphine-mediated upregulation of high mobility group box 1 in the spinal cord contributes to analgesic tolerance and hyperalgesia in rats. Neurotherapeutics 17, 722–742. 31879851 10.1007/s13311-019-00800-w PMC7283437

[R88] Raineki C, Chew L, Mok P, Ellis L, Weinberg J, 2016. Short- and long-term effects of stress during adolescence on emotionality and HPA function of animals exposed to alcohol prenatally. Psychoneuroendocrinology 74, 13–23. 27567117 10.1016/j.psyneuen.2016.08.015 PMC5159267

[R89] Relja B, Land WG, 2020. Damage-associated molecular patterns in trauma. Eur. J. Trauma Emerg. Surg. 46. 10.1007/s00068-019-01235-w PMC7427761 31612270

[R90] Rosen S, Ham B, Mogil JS, 2017. Sex differences in neuroimmunity and pain. J. Neurosci. Res. 95 (1–2). 10.1002/jnr.23831 27870397

[R91] Ruffaner-Hanson CD, , 2023. Prenatal alcohol exposure alters mRNA expression for stress peptides, glucocorticoid receptor function and immune factors in acutely stressed neonatal brain. Front. Neurosci. 17, 1203557. 37425005 10.3389/fnins.2023.1203557 PMC10326286

[R92] Saha PS, Mayhan WG, 2022. Prenatal exposure to alcohol: mechanisms of cerebral vascular damage and lifelong consequences. Adv. Drug Alcohol Res. 2, 10818. 38390614 10.3389/adar.2022.10818 PMC10880760

[R93] Sanchez JJ, Noor S, Davies S, Savage D, Milligan ED, 2017. Prenatal alcohol exposure is a risk factor for adult neuropathic pain via aberrant neuroimmune function. J. Neuroinflamm. 14, 254. 10.1186/s12974-017-1030-3 PMC5738192 29258553

[R94] Sanchez JJ, Sanchez JE, Noor S, , 2019. Targeting the β2-integrin LFA-1, reduces adverse neuroimmune actions in neuropathic susceptibility caused by prenatal alcohol exposure. Acta Neuropathol. Commun. 7. 10.1186/s40478-019-0701-y PMC6454692 30961664

[R95] Shen Y, Yang H, Wu D, 2022. NLRP3 inflammasome inhibitor MCC950 can reduce the damage of pancreatic and intestinal barrier function in mice with acute pancreatitis. Acta Cirúrg. Bras. 37. 10.1590/acb370706 PMC9633010 36327405

[R96] Silva Santos Ribeiro P, Willemen HLDM, Versteeg S, Martin Gil C, Eijkelkamp N, 2023. NLRP3 inflammasome activation in sensory neurons promotes chronic inflammatory and osteoarthritis pain. Immunother. Adv. 3, ltad022. 38047118 10.1093/immadv/ltad022 PMC10691442

[R97] Sn M, Ga B, Lr D, 2019. Fetal alcohol spectrum disorders: a review of the neurobehavioral deficits associated with prenatal alcohol exposure. Alcohol. Clin. Exp. Res. 43. 10.1111/acer.14040 PMC6551289 30964197

[R98] Staikopoulos V, Qiao S, Liu J, Song X, Yang X, Luo Q, Hutchinson MR, Zhang Z, , 2021. Graded peripheral nerve injury creates mechanical allodynia proportional to the progression and severity of microglial activity within the spinal cord of male mice. Brain Behav. Immun. 91. 10.1016/j.bbi.2020.11.018 33197546

[R99] Tapia-Abellán A, Angosto-Bazarra D, Martínez-Banaclocha H, , 2019. MCC950 closes the active conformation of NLRP3 to an inactive state. Nat. Chem. Biol. 15. 10.1038/s41589-019-0278-6 PMC7116292 31086329

[R100] Vijayaraj SL, , 2021. The ubiquitylation of IL-1β limits its cleavage by caspase-1 and targets it for proteasomal degradation. Nat. Commun. 12, 2713. 33976225 10.1038/s41467-021-22979-3 PMC8113568

[R101] Wan P, , 2022. AP-1 signaling pathway promotes pro-IL-1β transcription to facilitate NLRP3 inflammasome activation upon influenza a virus infection. Virulence 13, 502–513. 35300578 10.1080/21505594.2022.2040188 PMC8942419

[R102] Wan W, Cao L, Khanabdali R, , 2016. The emerging role of HMGB1 in neuropathic pain: a potential therapeutic target for neuroinflammation. J. Immunol. Res. 10.1155/2016/6430423 PMC4887637 27294160

[R103] Wang X, Loram LC, Ramos K, , 2012. Morphine activates neuroinflammation in a manner parallel to endotoxin. Proc. Natl. Acad. Sci. U. S. A. 109. 10.1073/pnas.1200130109 PMC3341002 22474354

[R104] Wang B, Wang LN, Wu B, , 2024. Astrocyte PERK and IRE1 signaling contributes to morphine tolerance and hyperalgesia through upregulation of lipocalin-2 and NLRP3 inflammasome in the rodent spinal cord. Anesthesiology 140. 10.1097/ALN.0000000000004858 38079113

[R105] Watkins LR, Hutchinson MR, Johnston IN, Maier SF, 2005. Glia: novel counter-regulators of opioid analgesia. Trends Neurosci. 28, 661–669. 16246435 10.1016/j.tins.2005.10.001

[R106] Wyns A, , 2023. The biology of stress intolerance in patients with chronic pain-state of the art and future directions. J. Clin. Med. 12. 10.3390/jcm12062245 PMC10057496 36983246

[R107] Xue S, , 2022. Receptor-Interacting protein kinase 3 inhibition relieves mechanical allodynia and suppresses NLRP3 inflammasome and NF-κB in a rat model of spinal cord injury. Front. Mol. Neurosci. 15, 861312. 35514432 10.3389/fnmol.2022.861312 PMC9063406

[R108] Yan J, McCombe PA, Pender MP, Greer JM, 2020. Reduced IκB-α protein levels in peripheral blood cells of patients with multiple sclerosis-a possible cause of constitutive NF-κB activation. J. Clin. Med. 9. 10.3390/jcm9082534 PMC7465818 32781504

[R109] Yang H, Zeng Q, Silverman HA, Gunasekaran M, George SJ, Devarajan A, Addorisio ME, Li J, Tsaava T, Shah V, Billiar TR, Wang H, Brines M, Andersson U, Pavlov VA, Chang EH, Chavan SS, Tracey KJ, 2021. HMGB1 released from nociceptors mediates inflammation. Proc. Natl. Acad. Sci. 118. 10.1073/pnas.2102034118 PMC8379951 34385304

[R110] Yao P-W, , 2019. Upregulation of tumor necrosis factor-alpha in the anterior cingulate cortex contributes to neuropathic pain and pain-associated aversion. Neurobiol. Dis. 130, 104456. 31028871 10.1016/j.nbd.2019.04.012

[R111] Yee YH, Chong SJF, Kong LR, Goh BC, Pervaiz S, 2021. Sustained IKKβ phosphorylation and NF-κB activation by superoxide-induced peroxynitrite-mediated nitrotyrosine modification of B56γ3 and PP2A inactivation. Redox Biol. 41, 101834. 33838472 10.1016/j.redox.2020.101834 PMC8056462

[R112] Yuan Z, , 2024. Effect of NLRP3 inflammasome induced astrocyte phenotype alteration in morphine tolerance. Front. Pharmacol. 15, 1434295. 39600361 10.3389/fphar.2024.1434295 PMC11588488

[R113] Zeng W, Wu D, Sun Y, , 2021. The selective NLRP3 inhibitor MCC950 hinders atherosclerosis development by attenuating inflammation and pyroptosis in macrophages. Sci. Rep. 11. 10.1038/s41598-021-98437-3 PMC8481539 34588488

[R114] Zhang P, Yang M, Chen C, Liu L, Wei X, Zeng S, , 2020. Toll-like receptor 4 (TLR4)/opioid receptor pathway crosstalk and impact on opioid analgesia, immune function, and gastrointestinal motility. Front. Immunol. 11. 10.3389/fimmu.2020.01455 PMC7360813 32733481

