# Supplementary figures and images for: Prenatal alcohol exposure promotes nerve injury-induced pathological pain following morphine treatment via NLRP3-mediated peripheral and central proinflammatory immune actions
Source: Brain Behav Immun. Author manuscript; Available in PMC 2025 Dec 8. (PMC12684907; doi:10.1016/j.bbi.2025.06.041)

(A) TLR4 mRNA in sciatic nerve

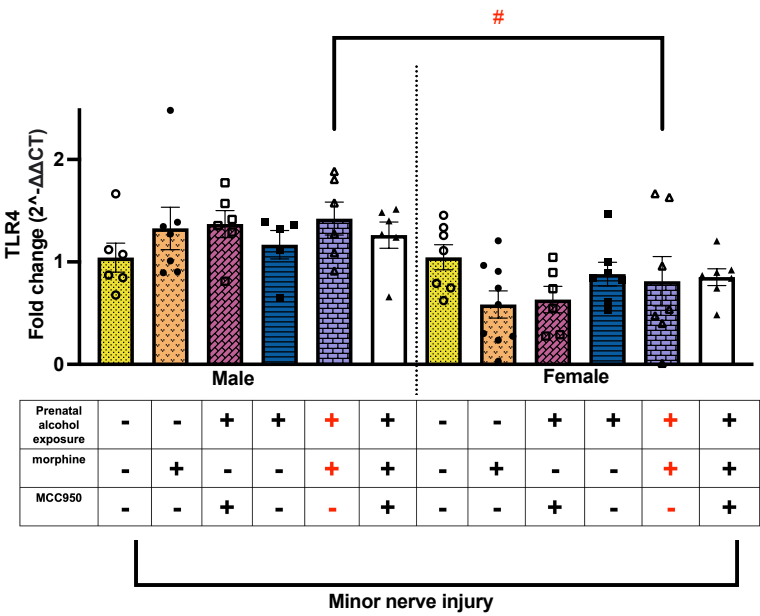

(B) IkBα mRNA in sciatic nerve

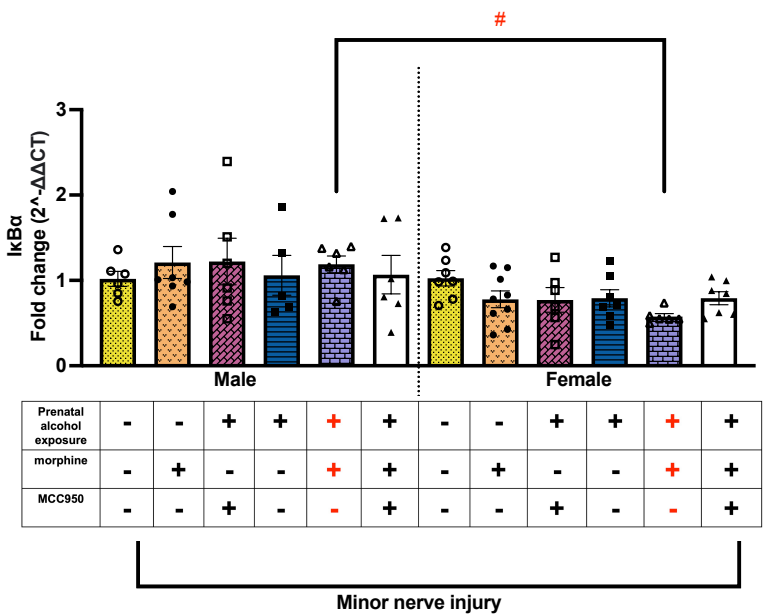

Supplement: MMC1 [file NIHMS2115567-supplement-MMC1.pdf]
